# Machine-Learning Classifier for Patients with Major Depressive Disorder: Multifeature Approach Based on a High-Order Minimum Spanning Tree Functional Brain Network

**DOI:** 10.1155/2017/4820935

**Published:** 2017-12-14

**Authors:** Hao Guo, Mengna Qin, Junjie Chen, Yong Xu, Jie Xiang

**Affiliations:** ^1^College of Computer Science and Technology, Taiyuan University of Technology, Taiyuan, China; ^2^National Laboratory of Pattern Recognition, Institute of Automation, The Chinese Academy of Sciences, Beijing, China; ^3^Department of Psychiatry, The First Hospital of Shanxi Medical University, Taiyuan, China

## Abstract

High-order functional connectivity networks are rich in time information that can reflect dynamic changes in functional connectivity between brain regions. Accordingly, such networks are widely used to classify brain diseases. However, traditional methods for processing high-order functional connectivity networks generally include the clustering method, which reduces data dimensionality. As a result, such networks cannot be effectively interpreted in the context of neurology. Additionally, due to the large scale of high-order functional connectivity networks, it can be computationally very expensive to use complex network or graph theory to calculate certain topological properties. Here, we propose a novel method of generating a high-order minimum spanning tree functional connectivity network. This method increases the neurological significance of the high-order functional connectivity network, reduces network computing consumption, and produces a network scale that is conducive to subsequent network analysis. To ensure the quality of the topological information in the network structure, we used frequent subgraph mining technology to capture the discriminative subnetworks as features and combined this with quantifiable local network features. Then we applied a multikernel learning technique to the corresponding selected features to obtain the final classification results. We evaluated our proposed method using a data set containing 38 patients with major depressive disorder and 28 healthy controls. The experimental results showed a classification accuracy of up to 97.54%.

## 1. Introduction

Resting-state functional magnetic resonance imaging (rs-fMRI) using blood oxygenation level-dependent (BOLD) signals as neurophysiological indicators can detect spontaneous low-frequency brain activity and has been successfully applied to the diagnosis of neuropsychiatric diseases such as schizophrenia [1–4], Alzheimer's disease [5–7], epilepsy [8–10], attention deficit hyperactivity disorder (ADHD) [11], and stroke [12, 13]. Resting functional brain network analysis helps clarify the mechanisms of neuropsychiatric disorders and has the potential to provide relevant imaging markers that may offer new perspectives for the diagnosis and evaluation of clinical brain diseases [2]. In traditional brain network analysis, it is assumed that the correlation between different brain regions does not change with time during rs-fMRI scanning. Networks constructed using methods based on this assumption are called low-order networks [14].

However, this assumption may lead researchers to overlook the dynamic interaction patterns between brain regions during the entire scan, which are essentially time-varying. Indeed, several recent studies have indicated that functional connectivity analyses can be rich in dynamic temporal information [15, 16]. High-order functional connectivity networks contain abundant dynamic time information, so this method has been proposed and applied in the diagnosis of brain diseases [14, 17].

The most common method for constructing a high-order functional network is the dynamic sliding window method, in which the whole rs-fMRI time series is divided into several time windows [18]. A low-order functional connectivity network is built in each time window, and then all the low-order networks are stacked. A clustering algorithm is performed to divide all relevant time series into several clusters. The average time series of each cluster is then taken as a new node, and the Pearson correlation coefficient is calculated between each node pair as the weight of connectivity [14].

In this method, clustering is employed to decrease the associated computational costs, and classification accuracy is greatly influenced by the randomness of the selection of initial clustering centers and the number of clusters. However, because the time series of all connectivities within each cluster are averaged, the network loses neurological interpretability.

In the present study, we used the minimum spanning tree method [19] to reduce the computational cost while preserving the core framework of networks. A classic approach in graph theory, this unbiased method greatly simplifies the network structure while preserving its core framework, thus avoiding the influences of network sparseness and other parameters on network structure. It also guarantees the network's neurological interpretability and has been widely used in previous studies [20–22].

Furthermore, the traditional feature extraction method in minimum spanning tree networks uses a quantifiable network with local features for classification of brain diseases, such as degree, clustering coefficient, minimum path length, and eccentricity [23, 24]. However, a clear shortcoming of this method was the chance that some of the useful topology information in the network (including connection patterns in the sample itself and the common connection patterns between the samples) would be lost, resulting in reduced classifier performance. Frequent subgraph mining technology was proposed to mine discriminative subgraph pattern features for machine-learning classification of brain diseases [25, 26]. Subgraph pattern features could account for the connection pattern information between multiple brain regions, but it was not sensitive to changes in single brain regions [27]. Therefore, both methods can lead to loss of sample information.

Here, we propose a novel feature extraction method that combines quantifiable local network features with subgraph pattern features. Specifically, we computed degree, eccentricity, and betweenness centrality of each brain region as local network features and extracted the subgraph features using a frequent subgraph mining method for a group of healthy controls (HC) and a group of people with major depressive disorder (MDD). Then a kernel function for each type of feature was constructed, namely, a vector kernel (local network features) and a graph kernel (subgraph features). Finally, the two kernel matrices were combined and a multikernel support vector machine was constructed as a classifier. The proposed method achieves better classification performance than traditional methods that use only a single type of feature.

## 2. Materials and Methods

### 2.1. Proposed Framework

**Figure 1:**
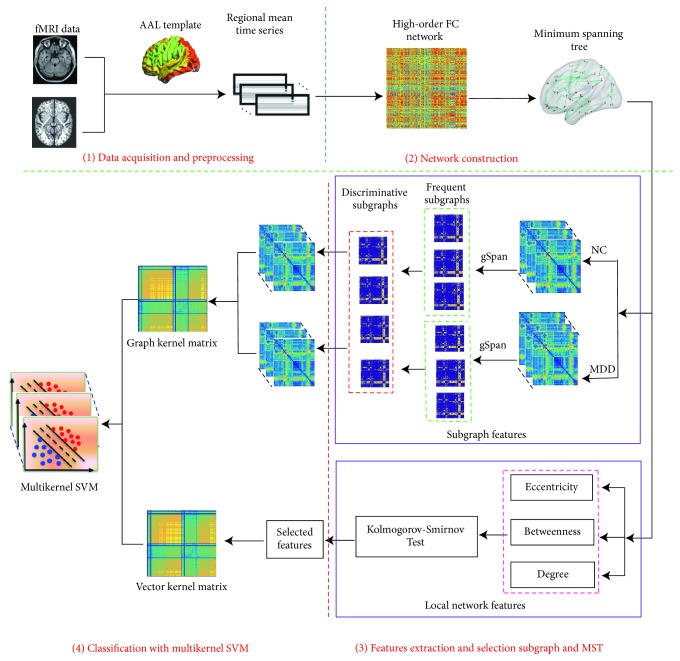
*Basic framework. *Illustration of the basic framework of the method used. (1) Data acquisition and preprocessing; (2) network construction (the high-order functional connectivity network is constructed first, followed by construction of the minimum spanning tree network); (3) feature extraction and selection (two types of feature are extracted and selected: one is to calculate quantifiable local network features (degree, betweenness centrality, and eccentricity) with the Kolmogorov–Smirnov test used for feature selection and the other is to mine frequent subgraphs from the HC and MDD groups and select the most discriminative subnetworks as the subgraph patterns); (4) classification model construction (kernel matrix is calculated for two types of feature and then the multiple-kernel support vector machine (SVM) is adopted to combine these heterogeneous kernels for distinguishing individuals with MDD from healthy controls). AAL, automated anatomical labeling; fMRI, functional magnetic resonance imaging; FC, functional connectivity; HC, healthy controls; MDD, major depressive disorder.

Figure 1 shows the flowchart of the proposed method, which includes four main steps: (1) data acquisition and preprocessing; (2) network construction, in which a high-order functional connectivity network is constructed first, followed by construction of a minimum spanning tree network; (3) feature extraction and selection, in which two types of feature are extracted and selected (the first is used to calculate quantifiable local network features (degree, betweenness centrality, and eccentricity) and uses the Kolmogorov–Smirnov test for feature selection, and the second is used to mine frequent subgraphs from the HC and MDD groups and selects the most discriminative subnetworks as the subgraph patterns); (4) construction of a classification model, in which the kernel matrix of the two types of feature is calculated. The multiple-kernel support vector machine (SVM) is adopted to combine the two heterogeneous kernels, enabling the distinction of individuals with MDD from healthy controls.

### 2.2. Data Acquisition and Preprocessing

The study was carried out in accordance with the recommendations of the medical ethics committee of Shanxi Province (reference number: 2012013). All subjects provided written informed consent in accordance with the Declaration of Helsinki. Twenty-eight healthy subjects and thirty-eight people with MDD underwent rs-fMRI in a 3T scanner (Siemens Trio 3-Tesla scanner, Siemens, Erlangen, Germany). Participant demographic information is shown in Table 1.

Data collection was completed at the First Hospital of Shanxi Medical University. Radiologists familiar with fMRI performed all scans. During each scan, the participant was asked to relax with their eyes closed and not think about anything in particular but to stay awake and avoid falling asleep. Each scan consisted of 248 contiguous echo-planar imaging (EPI) functional volumes (33 axial slices, repetition time (TR) = 2000 ms, echo time (TE) = 30 ms, thickness/skip = 4/0 mm, field of view (FOV) = 192 × 192 mm, matrix = 64 × 64 mm, and flip angle = 90°). The first 10 volumes in the time series were discarded to account for magnetization stabilization. See Supplemental Text S1 for detailed scanning parameters.

Data preprocessing was performed in SPM8 (http://www.fil.ion.ucl.ac.uk/spm/) with slice-timing and head-movement corrections. Two samples containing a translation of more than 3.0 mm and rotation of more than 3.0° were excluded from the final analysis of 66 samples. Functional images were normalized using the 12 parameters from the affine transformation and the cosine-based nonlinear transformation from the normalization of the anatomic image to the Montreal Neurological Institute (MNI) space. Additional normalization of the functional data sets to the SPM8 EPI template was then performed, and the data were resampled to a voxel size of 3 × 3 × 3 mm using a sinc interpolation. No smoothing kernel was applied to limit spurious local connectivity between voxels. Finally, we performed linear detrending and band-pass filtering (0.01–0.10 Hz) to reduce the effects of low-frequency drift and high-frequency physiological noise. Then, for each subject, the brain space of the fMRI images was parcellated into 90 regions of interest (ROIs) (45 in each hemisphere) based on the automated anatomical labeling (AAL) atlas [40], and each region was defined as a node in the network. Each regional mean time series was regressed against the average cerebral spinal fluid and white-matter signals as well as the six parameters from motion correction. The residuals of these regressions constituted the set of regional mean time series used for undirected graph analysis.

### 2.3. Construction of the High-Order Minimum Spanning Tree Network

#### 2.3.1. High-Order Functional Connectivity Network

**Figure 2:**
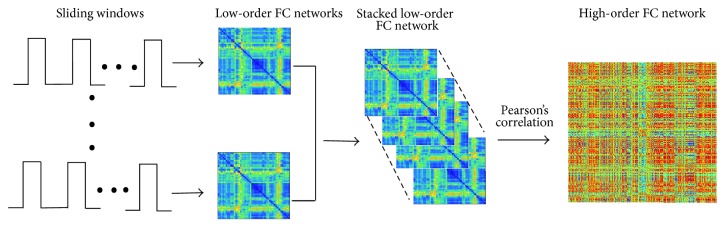
*High-order functional connectivity network construction flowchart*. (1) Partition the entire rs-fMRI time series into multiple overlapping segments of subseries by adopting a fixed-length sliding window; (2) construct temporal low-order FC networks in each time window; (3) stack all low-order FC networks for all subjects; (4) construct a high-order FC network for each subject, by taking the low-order FC as a new vertex and the pairwise Pearson's correlation coefficient between each pair of these new vertices as the weight. FC, functional connectivity.

A high-order functional connectivity network was constructed using a flowchart with the following steps (Figure 2): (1) partition the entire rs-fMRI time series into multiple overlapping segments of subseries by adopting a fixed-length sliding window; (2) construct temporal low-order functional connectivity networks in each time window; (3) stack together all low-order functional connectivity networks for all subjects; (4) construct a high-order functional connectivity network for each subject by taking the low-order functional connectivity as the new nodes and the pairwise Pearson correlation coefficient between each pair of nodes as the path weight.

To enable construction of a low-order functional connectivity network in each time window, we divided the whole time series *x*_*i*_^(*l*)^ into a number of overlapping subseries segments using the sliding time-window method. Specifically, if the length of the sliding window is *N* and the step size between two successive windows is *S*, let *x*_*i*_^(*l*)^ ∈ *R*^*N*^ denote the *k*th segment of the subseries extracted from *x*_*i*_^(*l*)^. The total number of segments generated by this approach is given by

(1) $$\begin{array}{c} K=\left\lfloor \;\frac{\left(M-N\right)}{s}\;\right\rfloor +1,\;1\leq k\leq K. \end{array}$$

The length of our sliding window was 90 and the step length was 1. For an illustration of the sliding window, see Supplemental Figure S1.

For the *L*th subject, the *k*th segment in the subseries for all ROIs can be expressed in matrix form as

(2) $$\begin{array}{c} x^{\left(l\right)}\left(\;k\;\right)=\left[\;x_{1}^{\left(l\right)}\left(\;k\;\right),x_{2}^{\left(l\right)}\left(\;k\;\right),\ldots ,x_{R}^{\left(l\right)}\left(\;k\;\right)\;\right]\in R^{N\times R}, \end{array}$$

where *R* is the total number of ROIs. Then, the entry for the *k*th temporal functional connectivity matrix for the *L*th subject *C*^(*l*)^(*k*) can be obtained by the Pearson correlation between the *i*th and *j*th ROIs. The *K* temporal functional connectivity networks for the *L*th subject can be established by taking {*y*_*ij*_^(*l*)^} as nodes and {*C*_*ij*_^(*l*)^(*k*)} as the weights of new edges, as per the following equation:

(3) $$\begin{array}{c} C_{L}^{\left(l\right)}\left(\;k\;\right)=\left(\;\left\{\;x_{i}^{\left(l\right)}\left(\;k\;\right)\;\right\},\left\{\;C_{ij}^{\left(l\right)}\left(\;k\;\right)\;\right\}\;\right)\;\left(k=1,2,\ldots ,k\right). \end{array}$$

In this way, it is possible to construct *K* dynamic temporal functional connectivity networks for each subject. For each ROI pair (*i*, *j*) for the *L*th subject, we can concatenate *C*_*ij*_^(*l*)^(*k*) to obtain a correlation time series:

(4) $$\begin{array}{c} y_{ij}^{\left(l\right)}=\left[\;C_{ij}^{\left(l\right)}\left(\;1\;\right),C_{ij}^{\left(l\right)}\left(\;2\;\right),\ldots ,C_{ij}^{\left(l\right)}\left(\;k\;\right)\;\right]\in R^{K}. \end{array}$$

We can then stack together all the dynamic temporal functional connectivity networks for each subject, as per (4).

The main goal of this article is to reveal the intrinsic relationship between the correlation time series {*y*_*ij*_^(*l*)^} and the dynamic temporal information contained within it. We calculated the Pearson correlation coefficient between each pair of correlation time series for each subject as follows:

(5) $$\begin{array}{c} H_{ij,pq}^{\left(l\right)}=corr\left(\;y_{ij}^{\left(l\right)},y_{pq}^{\left(l\right)}\;\right). \end{array}$$

Thus, the construction of a high-order functional connectivity network is achieved by taking {*y*_*ij*_^(*l*)^} as new nodes and {*H*_*ij*,*pq*_^(*l*)^} as the weights of new edges and then connecting nodes *y*_*ij*_^(*l*)^ and *y*_*pq*_^(*l*)^. The new high-order functional connectivity network can be represented as

(6) $$\begin{array}{c} G_{H}^{\left(l\right)}=\left(\;\left\{\;y_{ij}^{\left(l\right)}\;\right\},\left\{\;H_{ij,pq}^{\left(l\right)}\;\right\}\;\right). \end{array}$$

Therefore, {*H*_*ij*,*pq*_^(*l*)^} can be said to represent the high-order correlation, and the corresponding network *G*_*H*_^(*l*)^ represents the high-order functional connectivity network. The high-order correlation indicates the linear correlation strength between two correlation time series and reflects the interaction between up to four brain regions. Compared with the traditional network, the high-order functional connectivity network not only takes into account the time-varying characteristics of functional connectivity but also represents the more complex and abstract interaction patterns among brain regions.

#### 2.3.2. Minimum Spanning Tree

To further reduce the complexity of the high-order functional connectivity network, we constructed a minimum spanning tree. This is a weighted subnetwork (fully connected network) that connects all the nodes in the network without forming loops and has the minimum total weight of all possible spanning trees [24]. We constructed the minimum spanning tree based on the weighted network. Since we were interested in determining the strongest connection in the network, we used Kruskal's algorithm to obtain the strongest connection weights [41]. This algorithm first sorts the edges into descending weight order and then starts the construction of the minimum spanning tree from the largest-weight edge, adding the next largest-weight edge until all nodes *N* are connected in an acyclic subnetwork consisting of *M* = *N* − 1 edges. When the addition of an edge forms a loop, this edge is ignored. For more information regarding Kruskal's algorithm, see Supplemental Text S2.

### 2.4. Feature Extraction and Selection

After completion of the network, we extracted features of two different types: quantifiable local network features of the minimum spanning tree and subgraph patterns from frequent subgraph mining. We selected quantifiable local network features of the minimum spanning tree using the Kolmogorov-Smirnov test. For the connected patterns from frequent subgraph mining, we used discriminative scores to select the most discriminative subgraphs.

#### 2.4.1. Local Network Features and Selection Methods

We selected the local network properties of the minimum spanning tree (degree, betweenness centrality, and eccentricity) as features. We calculated the three properties of each node in the high-order minimum spanning tree network.  Table 2 gives the definition and formula of these three properties. We used multilinear regression analysis to assess the confounding effect of age, sex, and educational attainment on each network attribute. The independent variable was the mean of each network attribute (except for the degree, owing to its nature) and the dependent variables were age, sex, and educational attainment. The results showed no significant correlations between betweenness centrality, eccentricity, and corresponding variables (see Supplemental Table S1 for results).

We used the Kolmogorov-Smirnov test [42] to select the quantifiable local network features of the minimum spanning tree (*p* < 0.05). The results were then corrected using the Benjamini-Hochberg false positive rate (*q* = 0.05) [43].

#### 2.4.2. Frequent Subgraph and Discriminative Evaluation

(*1) Frequent Subgraph. *In this paper, subgraph pattern extraction was mainly based on frequent subgraph mining. The frequent subnetwork refers to the connected patterns that appear most often in the network [44]. The purpose of frequent subnetwork mining is to uncover the most frequent connected patterns (i.e., subnetworks) in the whole network [25]. We applied this algorithm to the HC and MDD groups. In the field of data mining, a large number of frequent subgraph mining methods have been proposed [45, 46], including a priori-based graph mining [47] and the frequent subgraph discovery algorithm [48]. Here, we used the well-known gSpan algorithm [49] to extract the frequent subnetworks from the functional connectivity network. Because of its high efficiency in graph traversal and subgraph mining, the gSpan algorithm has been widely applied in many research fields, including neural imaging [25–27].

The gSpan algorithm works as follows [50]. First, the gSpan constructs a new lexicographic order among graphs and maps each graph to a unique minimum depth-first search (DFS) code as its canonical label. Then, based on the lexicographic order, gSpan uses the DFS strategy to efficiently mine frequently connected subgraph patterns. In the present study, we termed the hierarchical search space of frequent subgraphs the “DFS code tree,” where each node in the tree represents a DFS code (i.e., subgraph). The *k* + 1th level subgraph is generated from the *k*th level subgraph (i.e., parent) by adding one frequent edge. Finally, all subgraphs with nonminimal DFS codes are pruned to avoid redundant candidate generations. In subgraph mining, the number of subgraphs is mainly controlled by frequency. Given a set of graphs, *G*, the frequency of a subgraph *g*_*s*_ is defined as

(7) $$\begin{array}{c} fq\left(\;g_{s}\;|\;G\;\right)=\frac{\left|g_{s}\;\;is\;\;a\;\;subgraph\;\;of\;\;g,\;\;g\in G\right|}{G}. \end{array}$$

The DFS lexicographic order used in frequent subgraph mining and the gSpan algorithm are described in detail in Supplemental Text S3.

(*2) Discriminative Evaluation. *The discriminative subnetwork can be used as a feature for classification [51], but it is worth noting that gSpan is only used for mining the frequent subgraph, which, by itself, has no discriminative power. For information on the discriminative capabilities of different subgraphs, see Supplemental Figure S2. However, some of the frequent subnetworks may have less discriminative information for classification. To address this problem, we selected the most discriminative subnetworks from the frequent subnetworks using subgraph discriminative scores (which express subgraph frequency differences) [27]. This strategy is called frequent-scoring feature selection. In the present study, the method involved choosing the same number of frequent subgraphs from the HC and MDD groups, calculating and sorting the discriminative scores of frequent subgraphs, and selecting the top *k* subnetworks with higher discriminative scores. Thus, 2*∗k* discriminative subnetworks are selected. For the given graphs *G*_*P*_ and *G*_*n*_, *G*_*P*_ = {*g*_*p*1_, *g*_*p*2_,…, *g*_*pm*_} refers to the set of frequent subgraphs for all positive samples and *G*_*n*_ = {*g*_*n*1_, *g*_*n*2_,…, *g*_*nm*_} refers to the set of all frequent subgraph features for negative samples. The discriminative scores *S*(*g*_*s*_) of subgraph *g*_*s*_ can be calculated as

(8) $$\begin{array}{c} S\left(\;g_{s}\;\right)=\left|\;f_{q}\left(\;g_{s}\;|\;G_{P}\;\right)-f_{q}\left(\;g_{s}\;|\;G_{n}\;\right)\;\right|. \end{array}$$

The discriminative score of a subgraph pattern *g*_*s*_ is simply defined as the difference between its positive frequency and negative frequency. A larger score reflects a larger difference between the patterns in the two groups. *S*(*g*_*s*_) = 1 indicates that the subgraph *g*_*s*_ exists in all graphs for the HC group and that there is no such pattern in any graph for the MDD group. *S*(*g*_*s*_) = −1 indicates that the subgraph *g*_*s*_ exists in all graphs for the MDD group and that there is no such pattern in any graph for the HC group.

### 2.5. Construction of Classification Model

The classification model chosen in this paper is a multikernel SVM. Recent studies on multikernel learning have shown that the integration of multiple kernels can significantly improve classification and enhance interpretability of results [52]. Generally, the integration of the kernel is achieved by linear combination of multiple kernels:

(9) $$\begin{array}{c} k\left(\;x,y\;\right)=\overset{M}{\underset{i=1}{\sum }}a_{i}k_{i}\left(\;x,y\;\right)\;s.t\;\;\overset{M}{\underset{i=1}{\sum }}a_{i}=1, \end{array}$$

where *k*_*i*_(*x*, *y*) is a basic kernel built for subjects *x* and *y*, *M* is the number of kernel matrices required, and *a*_*i*_ is the nonnegative weighting parameter.

A graph kernel can be regarded as a group of similarities between a pair of subjects. The brain network data is mapped from the original network space to the feature space, and the similarity between the two brain networks is further measured by comparing their topology. In this study, we used the Weisfeiler-Lehman subtree, based on the Weisfeiler-Lehman isomorphism test [53], to measure the topological similarity between paired connectivity networks. This type of graph kernel can effectively capture topological information from graphs and improve performance. Given two graphs, the basic process of the Weisfeiler-Lehman test is as follows: if the two graphs are unlabeled (i.e., the nodes of the graph have not been assigned labels), each node is first labeled with the number of edges that are connected to that node. Then, at each iteration step, the label of each node is updated based on its previous label and the labels of its neighbors. That is, the sorted set of updated node labels for each node is compressed such that it contains new and shorter labels. This process iterates until the node label sets are identical or the number of iterations reaches its predefined maximum value. For a detailed description of the Weisfeiler-Lehman isomorphism test and pseudocode, see Supplemental Text S4.

As this study involves two different types of kernel (vector-based kernels and graph kernels), a normalization step must be performed individually before combining them. This normalization step can be accomplished using the following formula:

(10) $$\begin{array}{c} k^{*}\left(\;x,y\;\right)=\frac{k\left(x,y\right)}{\sqrt{k\left(x,x\right)k\left(y,y\right)}}. \end{array}$$

Note that, unlike the previous multikernel learning method, in which the weighting parameters *a*_*i*_ are jointly optimized together with other classifier parameters, in this study, the optimal weighting parameters *a*_*i*_ are determined via a grid search of the training data. Once the optimal weighting parameters *a*_*i*_ are obtained, the multikernel learning-based classifier can be naturally embedded into the conventional single-kernel classifier framework. In this paper, we selected the SVM as the classifier framework.

As described above, we used multikernel learning methods to perform classification. As different types of kernels represent different properties of the network, we combined multiple features through multikernel learning. Specifically, the vector-based kernel describes the correlation between pairwise brain regions according to degree, betweenness centrality, and eccentricity, and the graph-based kernel describes the topological information contained in the whole network.

## 3. Results

We performed two types of feature extraction on the constructed network. The first involved the quantifiable local network features, namely, degree, betweenness centrality, and eccentricity. The second involved the extraction of discriminative subgraph patterns from the HC and MDD groups.

### 3.1. Abnormal Functional Connectivities

The high-order functional connectivity network was 4005*∗*4005, so there were 4004 edges in the high-order minimum spanning tree network. After constructing the network, we analyzed three kinds of traditional quantifiable network properties. We selected the high-order functional connectivities of at least two network properties with *p* < 0.05 (false discovery rate corrected). We obtained 40 abnormal functional connectivities in total, encompassing a total of 42 abnormal regions (Table 3). All 40 significant abnormal functional connectivities and frequency-corresponding nodes are shown in Supplemental Figure S3. These significant regions were concentrated in the limbic-cortical networks (left anterior cingulate and paracingulate gyri; bilateral median cingulate and paracingulate gyri; right posterior cingulate gyrus; bilateral caudate nucleus; bilateral lenticular nucleus; bilateral putamen; bilateral thalamus; bilateral hippocampus; bilateral parahippocampal gyrus; and bilateral amygdala), frontal lobe (bilateral precentral gyrus; bilateral dorsolateral superior frontal gyrus; bilateral superior frontal gyrus, orbital part; right middle frontal gyrus; bilateral middle frontal gyrus, orbital part; bilateral inferior frontal gyrus, triangular part; and bilateral inferior frontal gyrus, opercular part), temporal lobe (right temporal pole: middle temporal gyrus; left Heschl gyrus), and cuneus (bilateral cuneus; bilateral lingual gyrus; bilateral precuneus; right postcentral gyrus; left calcarine fissure; and surrounding cortex).

We selected the top 10 brain regions with the most significant differences in terms of frequency (Table 4). These mainly included the bilateral temporal pole: middle temporal gyrus; left superior frontal gyrus, orbital part; left thalamus; right lenticular nucleus; putamen; left lingual gyrus; right cuneus; left posterior cingulate gyrus; and right dorsolateral superior frontal gyrus.

### 3.2. Frequent Subgraph Patterns

We analyzed the frequent discriminative subnetworks. We mined two sets of frequent subnetworks from the functional connectivity networks of the MDD and HC groups, with respective frequencies fq = 0.286 and fq = 0.211. Specifically, we mined 4057 subgraphs from the HC group and 4078 from the MDD group. For statistical information regarding the number of edges in the subgraphs, see Supplemental Table S3. We calculated the discriminative scores of the frequent subgraphs and found 16 subgraphs for the HC group and 37 for the MDD group. To ensure that the features were balanced, we selected 16 discriminative subnetworks to assess the subgraph patterns from the two groups.

To analyze the connected patterns, connections in the 16 subgraph connected patterns for each of the HC and MDD groups were merged with the subgraph in Supplemental Figure S4. By analyzing the subgraphs of the HC and MDD groups, we found some nodes that were significantly different between the two groups. These significantly different nodes were mainly concentrated in the bilateral lenticular nucleus: the putamen; bilateral lingual gyrus; bilateral amygdala; bilateral thalamus; bilateral median cingulate and paracingulate gyri; right posterior cingulate gyrus; bilateral cuneus; left anterior cingulate and paracingulate gyri; right superior frontal gyrus, orbital part; right middle frontal gyrus; right temporal pole; middle temporal gyrus; left precentral gyrus; right lenticular nucleus; pallidum; and so forth.

We also analyzed these significantly different brain regions and ranked them according to the frequency in which they appeared in the HC and MDD groups. The significantly different brain regions and the frequency-corresponding nodes are given in Supplemental Figure S5. We selected the top 10 regions as the most discriminative (Table 5).

### 3.3. Classification Results

We evaluated the classification performance of the proposed method by measuring classification accuracy, sensitivity, specificity, and area under the curve (Table 6). Table 6 also compares the classification performance of the partial correlation functional connectivity network, Pearson functional connectivity network, high-order functional connectivity network, and frequent subgraph mining methods. The results indicate that our proposed method achieves good results in terms of classification accuracy, sensitivity, specificity, and area under the curve.

**Figure 3:**
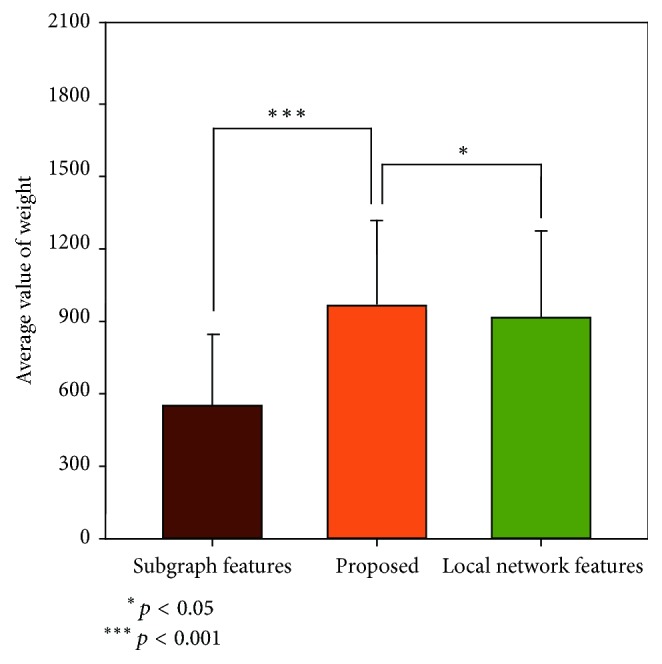
*Average weight of different types of feature.* Statistical analysis of the average weight for subgraph pattern features, minimum spanning tree of quantifiable local network features, and features used in this study. The average weight of subgraph pattern features was 550.31, the minimum spanning tree of quantifiable local network features was 915.42, and that for both feature types together was 945.16. The combination of the two different types of features had the greatest weight.

**Figure 4:**
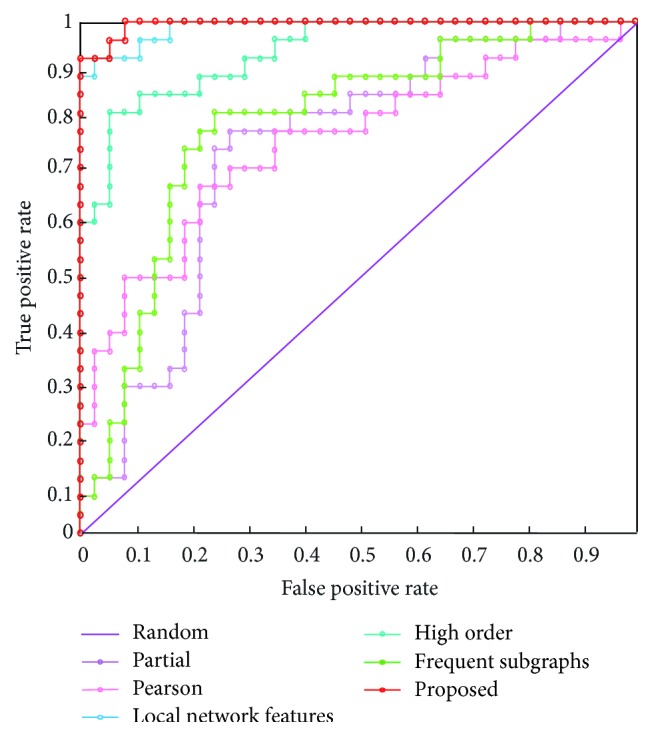
*ROC curves of the different methods.* Receiver operating characteristic (ROC) curves of the proposed method, the partial correlation network, the Pearson correlation network, the higher-order functional connectivity network, and the method using only subgraph patterns as features and quantifiable local network features. The proposed method has the greatest ROC.

Specifically, to compare the method proposed in this paper with those used previously, we constructed partial and Pearson correlation networks and a high-order functional connectivity network without minimum spanning tree analysis (see Supplemental Text S5 for the detailed information of other contrast networks). In addition, we used a high-order minimum spanning tree network for assessing quantifiable local network features and subgraph patterns as features. Our experimental results showed that the proposed method of classification performed significantly better than the partial correlation network, Pearson correlation network, and high-order functional connectivity network and also better than the method in which only the quantifiable local network features and subgraph pattern features were assessed. This illustrates the potential for the integration of the two different types of features to significantly improve classification performance. We used the Relief method [49] to calculate the average weights of subgraph pattern features, the minimum spanning tree of quantifiable local network features, and both types of features combined (Figure 3). The average weight of the subgraph pattern features was 550.31, that of the minimum spanning tree of the quantifiable local network features was 915.42, and that for both feature types together was 945.16.  Figure 4 shows the receiver operating characteristic curve of the proposed method, the partial correlation network, the Pearson correlation network, the higher-order functional connectivity network, and the approach with only subgraph patterns as features and quantifiable local network features. These results indicate that our proposed method clearly improved classification performance.

## 4. Discussion

### 4.1. Abnormal Brain Regions

We extracted quantifiable local network features and frequent subgraph features to explore brain regions with significantly abnormal connectivities between the HC and MDD groups. By calculating the quantifiable local network features, we were able to obtain 40 significant abnormal connectivities, involving 42 brain regions. Then, we selected the top 10 most frequently implicated brain regions, as those were the most significantly different between the two groups. Consistent with previous studies, these regions included the bilateral temporal pole: the middle temporal gyrus; the left superior frontal gyrus, orbital part; the right lateral dorsolateral frontal gyrus; the left thalamus; the right putamen; the left lingual gyrus; the right cuneus; and the left posterior cingulate gyrus.

The present results are consistent with our previous findings. Ma et al. [54] adopted voxel-based morphometry to investigate brain regions with gray-matter abnormality in patients with treatment-resistant depression and in those with treatment-responsive depression. They found that patients in both groups showed clear gray-matter abnormalities in the right temporal pole of the temporal gyrus, specifically the middle temporal gyrus. Qiu et al. [55] examined cortical thickness and surface area in first-episode, treatment-naïve, mid-life MDD and observed a significant increase in gray-matter volume in the left superior frontal gyrus, left thalamus, and right cuneus. Sacchet et al. [56] obtained whole-brain T1-weighted images in their own HC and MDD groups and evaluated gray-matter volumes in the basal ganglia (specifically the caudate nucleus, lentiform pallidum, and putamen). They reported that gray-matter volumes in the bilateral lenticular nucleus and putamen were significantly different between patients with depression and healthy controls. Jung et al. [57] used voxel-based morphometry to detect structural changes in healthy subjects and patients with depression who underwent 8 weeks of antidepressant treatment. The results showed significantly different gray-matter volume in the left lingual gyrus between the two participant groups. Fang et al. [58] measured spontaneous whole-brain homodynamic responses using amplitude of low-frequency fluctuation (ALFF) and fractional ALFF (fALFF) and found that the ALFF and fALFF decreased in the left posterior cingulate gyrus, the right cuneus, and the superior frontal gyrus after depression treatment. Cotter et al. also found abnormalities in the dorsal prefrontal cortex of patients with MDD [59].

In the present study, frequent subgraph mining revealed a total of 32 discriminative patterns (16 in the HC group and 16 in the MDD group). We found 19 common brain regions in the 32 connected patterns in the two groups. According to the frequency of each region in the connected patterns, we selected the 10 most discriminative brain regions. These included the bilateral lenticular nucleus putamen, bilateral lingual gyrus, bilateral amygdala, left median cingulate paracingulate gyri, right posterior cingulate gyrus, and bilateral thalamus. Anand et al. [31] studied the differences between limbic-cortical activities and connections in patients with MDD versus HCs. They found significant differences between patients and controls in the bilateral anterior cingulate cortex, bilateral amygdala, and bilateral thalamus. The top 10 most frequent regions in our study included the amygdala, which is part of the limbic system and is involved in the formation of emotional behavior, spontaneous activity, and endocrine integration processes. Previous studies [60–62] have indicated that the amygdala plays a significant role in the pathogenesis of depression. Veer et al. [29] used independent component analysis to assess rs-fMRI data from 19 medication-free patients with a recent diagnosis of MDD (within 6 months prior to inclusion) and no comorbidity and 19 age- and gender-matched controls. They found decreased activation in the bilateral amygdala, which is associated with emotional behavior, the frontal lobe, which is associated with attention and working memory, and the lingual gyrus, which is related to visual processing. The other discriminative brain regions that we identified via frequent subgraph mining are also consistent with previous results, such as the bilateral lenticular putamen [56], the left median cingulate and paracingulate gyri [36], and the right posterior cingulate gyrus [57].

Three of the brain regions with the most significant differences were obtained by two analysis methods: quantifiable local network features and discriminative subgraph patterns. These included the right lenticular nucleus (putamen), left lingual gyrus, and left thalamus. The right lenticular nucleus (putamen) and the left thalamus are key regions of the limbic-cortical circuit and the default network. Meng et al. [63] suggested that reward-related dysfunction of the right putamen within a striatum-centered limbic-cortical circuit may inhibit learning related to appreciating and enjoying positive life experiences, which is critical for depression recovery. As the thalamus makes a critical connection between the amygdala and the prefrontal cortex, it is well positioned for involvement in MDD pathophysiology. Similarly, the right lingual gyrus is a key region of the visual network. Jung et al. reported that the volume of the lingual gyrus is associated with neuropsychological features of depression [57]. The lingual gyrus plays an important role in visual processing. Therefore, the results of this study may be helpful in the search for biomarkers of MDD.

### 4.2. Classification Result Analysis

To study dynamic changes in functional connectivities between brain regions, Chen et al. used sliding time windows to construct a high-order functional connectivity network that can be used for classification [14]. Their method has a high accuracy for diagnosing mild cognitive impairment (MCI). To show that features obtained from subgraph patterns can better reflect the topological information among brain regions, Du et al. adopted frequent subgraph mining technology to mine frequent subnetworks in fMRI data from people with ADHD. They used a frequent-scoring feature selection method to choose discriminative subnetworks and kernel principal component analysis to extract features, before using LIBSVM (library for support vector machines) for classification [26]. Wang et al. also used frequent subgraph mining techniques to mine discriminative subnetworks that were based on fMRI data from people with MCI [27]. They combined traditional quantifiable properties with local clustering coefficients as features and then used a multikernel SVM for classification. And Fei et al. used frequent subgraph mining techniques combined with a discriminative subnetwork mining algorithm [25]. They then used a graph kernel-based SVM for classification. Their results showed that frequent subgraph patterns are highly accurate as features of classification.

Table 6 compares the accuracy, specificity, sensitivity, and area under the curve of the methods used in the present study. Different methods can produce different results using the same data set, and similar methods can produce different results with different data sets. Therefore, we constructed a partial correlation network, Pearson correlation network, and high-order functional connectivity network with the same data set. Our method for constructing a high-order minimum spanning tree network is superior to the other networks (Table 6). Compared with the traditional method, the high-order minimum spanning tree network can reveal stronger and more complex interactions between brain regions and thus may significantly improve the diagnostic accuracy rate in patients with MDD. Likewise, construction of a high-order minimum spanning tree functional connectivity network may result in better extraction of information regarding the interactions between brain regions in original rs-fMRI time series.

We independently classified quantifiable local network features and subgraph patterns as features on the same data set. Regardless of classification accuracy, sensitivity, specificity, or area under the curve, the classification result produced by the method proposed in this study performed better than an analysis with only quantifiable local network features or with only the subgraph pattern as features (Table 6). We used the Relief method to calculate the average weights for subgraph pattern features, quantifiable local network features, and both types of feature combined. As a result, our proposed method obtained the highest average weights. The Relief algorithm is a feature-weighting algorithm in which weights are continuously adjusted to show correlation between features, until the feature with the largest weight can be identified. Because this algorithm has high efficiency and can be used to make an accurate selection of discriminative features, it has been widely used in many fields, including biomedicine [64]. Given the results from the classification and feature analysis using the Relief method, the combination of quantifiable local network features and subgraph patterns as features appears to effectively reflect the information contained in a single brain region, while simultaneously reflecting the topological information contained in multiple brain regions. Combining these two different types of feature is likely to substantially improve diagnostic accuracy for patients with MDD.

### 4.3. Influence of Frequency on Graph Features

In this experiment, we mined frequent subnetworks from the functional connectivity network, which was constructed using data from the HC and MDD groups. This construction involved the selection of frequency, which can control the number of selected graph features. However, the high-order functional connectivity network indicates that there exists a temporal correlation between different low-order, dynamic functional connectivity networks. Thus, the size of the network can reach 4005*∗*4005. Even if the intercept associated with sparsity is very small (0.1 or 0.05), the size of the network can reach tens of thousands of edges, and the data for each subject will also have tens of thousands of edges. Hence, the number of subgraphs will be larger when mining frequent subgraphs, which is not conducive to the selection or analysis of subgraph features. Therefore, we constructed the minimum spanning tree network after constructing the high-order functional connectivity network. This method guarantees the integrity of the topological information associated with the high-order functional connectivity network and reduces the complex scale of the network. However, each subject in the minimum spanning tree network has only 4004 edges, which account for only 0.02% of high-order functional connectivity. Thus, frequent subgraph mining frequency must not be too big; if it is, subgraph pattern mining will not be possible. In contrast, if the frequency selection is too small, the subgraph patterns may be too large, increasing the chance of abandoning a large number of discriminative subnetwork patterns in the process of frequent subgraph mining. In the present study, we selected the frequencies of the HC and MDD groups as 0.29 and 0.21, respectively. We left the minimum spanning tree quantifiable local network features unchanged and only changed the features of the subgraph patterns. The different frequencies of the HC and MDD groups were used for classification. The classification results were optimal when the frequencies were 0.29 and 0.21 for the HC and MDD groups, respectively (Table 7).

### 4.4. Influence of Optimal Weighted Parameters *a*_*i*_ on Classification

**Figure 5:**
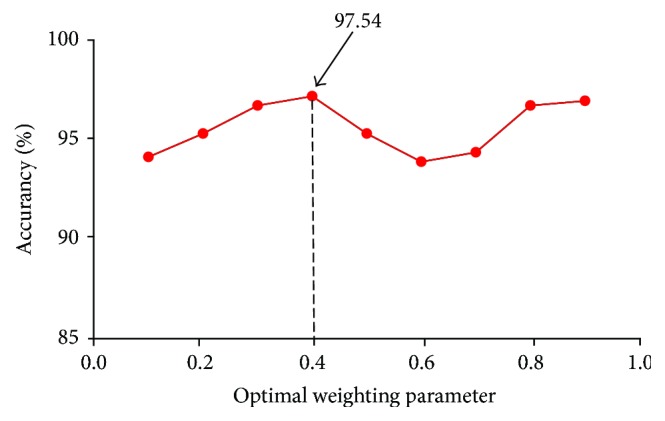
*Classification accuracy of different optimal parameters.* Optimal parameters were selected from a range of 0-1; step size, 0.1. Accuracy of classification with different optimal parameters was 94%–97%. Classification accuracy was greatest (97.54%) when the optimal parameter was 0.4.

Multikernel SVMs are widely adopted in neuroimaging classification [27]. Optimizing the weighting parameter *a*_*i*_ is very important in such classification, and optimal parameter selection will affect the classification results. We tested optimal parameters from 0 to 1 with a step size of 0.1.  Figure 5 shows the classification accuracy of different parameters. Classification accuracy was 94%–98% when different optimal parameters were used and was highest (97.54%) when the optimal parameter was 0.4.

## 5. Conclusion

The high-order functional connectivity network is relatively large, making it computationally expensive to use certain elements of complex network or graph theory to calculate topological properties. In the construction of a network, previous classification methods are based on local network features, so some useful network topology information may be lost. To address this, we proposed and tested the high-order minimum spanning tree to reduce computational consumption. We combined quantifiable local network features with discriminative subgraph patterns as features and then used a multikernel SVM for classification. The results showed that the high-order minimum spanning tree functional connectivity network could reflect dynamic changes in functional connectivities between brain regions. Additionally, the high-order network takes into account time-varying characteristics, such that the functional connectivity can reflect stronger and more complex interactions among more brain regions. The consistency of the results from the two different types of feature, that is, quantifiable local network features and frequent discriminative subgraph patterns, indicates that the detected significant differences between brain regions were consistent. More importantly, compared with traditional methods, the proposed method appears to offer better classification performance and thus may greatly improve the accuracy of MDD diagnosis. In future work, we plan to explore the impact of these functional connectivities and the relationship between the various ROIs, with the aim of further improving classification performance and better explaining pathology.

## Figures and Tables

**Table 1:** Subject demographics and clinical characteristics.

	HC	MDD	*p* values
Age	17–51 (26.6 ± 9.4)	17–49 (28.4 ± 9.68)	0.44^a^
Sex (male/female)	13/15	15/23	0.57^b^
Handedness (R/L)	28/0	38/0	
HAMD	N/A	15–42 (22.8 ± 13.3)	

Data are minimum–maximum (mean ± standard deviation). HAMD, 24-item Hamilton scale; ^a^two-sample, two-tailed *t*-test; ^b^two-tailed Pearson's chi-squared test.

**Table 2:** Definitions and formulae of minimum spanning tree network properties.

Concept	Explanation	Formula
Degree	Number of links for a given node	$k_{i}=\underset{j\in N}{\sum }a_{ij}$

Eccentricity	Longest shortest path from a reference node to any other node in the minimum spanning tree	Ecc(*v*) = max⁡{*d*(*u*, *v*)}

Betweenness centrality	Fraction of all shortest paths that pass through a particular node	BCi=1(n-1)(n-2)∑h,j∈Nh≠j,h≠iρhj(i)ρhj

*a*_*ij*_, connection between *i* and *j*; *d*(*u*, *v*), shortest path from *u* to *v*; *ρ*_*hj*_, number of shortest paths between* h* and* j*; *ρ*_*hj*_^(*i*)^, number of shortest paths between *h* and *j* which passthrough *i*.

**Table 3:** The 40 functional connectivities and associated statistical significance.

Number	FC connectivities		*p* values		
			Betweenness	Degree	Eccentricity
(1)	PreCG.L	SFGdor.R	**0.0135**	**0.0110**	0.0506
(2)	PreCG.L	PUT.R	**0.0199**	0.6648	**0.0303**
(3)	PreCG.R	ORBsup.L	0.8049	**0.0379**	**0.0379**
(4)	SFGdor.R	CUN.L	**0.0491**	**0.0412**	0.6491
(5)	SFGdor.R	TPOmid.R	**0.0379**	0.2216	**0.0122**
(6)	ORBsup.L	PUT.L	0.7558	**0.0396**	**0.0026**
(7)	ORBsup.L	PCL.R	**0.0252**	0.5730	**0.0252**
(8)	ORBsup.L	CAU.R	0.8504	**0.0135**	**0.0135**
(9)	ORBsup.R	DCG.R	**0.0252**	0.2943	**0.0252**
(10)	MFG.L	STG.L	**0.0077**	**0.0470**	0.4780
(11)	MFG.R	PCG.L	**0.0276**	0.4262	**0.0362**
(12)	MFG.R	PCG.L	**0.0135**	**0.0135**	0.1226
(13)	PCUN.L	CAU.R	**0.0149**	0.8049	**0.0149**
(14)	IFGtriang.R	ORBmid.R	**0.0009**	**0.0129**	0.3289
(15)	ORBinf.R	LING.L	0.4039	**0.0063**	**0.0173**
(16)	ORBinf.R	ACG.R	**0.0105**	**0.0362**	**0.0105**
(17)	AMYG.R	LING.R	**0.0190**	0.7431	**0.0029**
(18)	AMYG.L	TPOmid.R	**0.0029**	**0.0264**	0.6648
(19)	SFGmed.L	ACG.L	0.1264	**0.0470**	**0.0264**
(20)	THA.R	ACG.L	**0.0470**	**0.0036**	0.9172
(21)	DCG.L	THA.L	0.1752	**0.0210**	**0.0077**
(22)	ACG.R	LING.R	**0.0209**	**0.0167**	**0.0276**
(23)	DCG.R	PUT.R	**0.0029**	**0.0252**	0.1029
(24)	PHG.L	TPOmid.R	**0.0180**	0.4377	**0.0252**
(25)	AMYG.L	HES.L	**0.0470**	**0.0095**	0.5470
(26)	AMYG.R	MTG.R	**0.0368**	0.1368	**0.0095**
(27)	CUN.L	ORBmid.L	**0.0379**	**0.0243**	**0.0379**
(28)	CUN.R	PUT.R	**0.0157**	**0.0241**	0.3930
(29)	CUN.L	HES.L	**0.0209**	**0.0053**	0.7683
(30)	PCG.R	LING.R	**0.0033**	0.1693	**0.0173**
(31)	ORBsup.R	TPOmid.R	**0.0241**	**0.0122**	**0.0241**
(32)	PCG.R	THA.L	0.8282	**0.0396**	**0.0122**
(33)	PCG.R	TPOmid.L	**0.0396**	**0.0258**	0.7558
(34)	PreCG.R	THA.L	**0.0105**	0.8049	**0.0379**
(35)	HIP.L	PAL.L	0.0662	**0.0382**	**0.0289**
(36)	HIP.L	HES.L	0.2379	**0.0431**	**0.0379**
(37)	PCL.R	PAL.L	**0.0317**	0.8282	**0.0317**
(38)	PUT.L	TPOmid.R	**0.0004**	**0.0338**	**0.0027**
(39)	PAL.L	THA.R	0.1824	**0.0048**	**0.0396**
(40)	ROL.R	ITG.R	**0.0095**	**0.0432**	0.5220

Values in bold indicate significance (*p* < 0.05). For all brain region abbreviations, see Supplemental Table S2.

**Table 4:** Top 10 regions of interest in the minimum spanning tree.

Number	ROIs name	Citations
(1)	Temporal_Pole_Mid_R	Li et al. 2005 [28]
(2)	Frontal_Sup_Orb_L	Veer et al., 2009 [29]
(3)	Thalamus_L	Greicius et al., 2007 [30]
(4)	Pallidum_L	Anand et al., 2005 [31]
(5)	Lingual_L	Veer et al., 2009 [29]
(6)	Cuneus_R	Tao et al., 2013 [32]
(7)	Cingulum_Post_L	Wang et al., 2016 [33]
(8)	Frontal_Sup_R	Wang et al., 2016 [33]
(9)	Putamen_R	Anand et al., 2005 [31]
(10)	Temporal_Pole_Mid_L	Yue et al., 2013 [34]

For all brain region abbreviations, see Supplemental Table S2.

**Table 5:** Top 10 regions of interest in the subgraph patterns.

Number	ROIs name	Citations
(1)	Putamen_R	Anand et al., 2005 [31]
(2)	Lingual_R	Veer et al., 2009 [29]
(3)	Amygdala_L	Yue et al., 2013 [34]
(4)	Lingual_L	Veer et al., 2009 [29]
(5)	Cingulum_Mid_L	Sheline et al., 2010 [35]
(6)	Thalamus_L	Greicius et al., 2007 [30]
(7)	Thalamus_R	Greicius et al., 2007 [30]
(8)	Cingulum_Post_R	Wang et al., 2016 [33]
(9)	Putamen_L	Anand et al., 2005 [31]
(10)	Amygdala_R	Yue et al., 2013 [34]

For all brain region abbreviations, see Supplemental Table S2.

**Table 6:** Comparison of classification results from different methods.

Method	Research	Disease	Accuracy	Sensitivity	Specificity	AUC
Partial FC	Guo et al., 2013 [36]	MDD	86.01%	-	-	-
	Qiao et al., 2016 [37]	MCI	89.01%	86.67%	91.30%	-
	This study	MDD	63.06%	50.56%	87.37%	71.02%

Pearson FC	Wong et al., 2012 [38]	MDD	63.00%	40.00%	83.00%	-
	Liu et al., 2015 [39]	SAD	82.50%	85.00%	80.00%	-
	This study	MDD	66.67%	46.43%	81.58%	74.46%

High-order FC	Chen et al., 2016 [14]	MCI	88.14%	86.21%	90.00%	92.99%
	This study	MDD	92.51%	88.51%	93.19%	92.83%

Frequent subgraph	Du et al., 2016 [26]	ADHD	94.91%	93.22%	96.94%	96.90%
	Fei et al., 2014 [25]	MCI	97.30%	-	-	95.83%

Frequent and local cluster coefficient	Wang et al., 2014 [27]	MCI	97.27%	-	-	92.00%

High-order MST FC	Subgraph features	MDD	73.32%	80.36%	67.58%	75.67%
	Minimum spanning tree features	MDD	94.04%	98.26%	92.50%	97.84%
	Proposed	MDD	97.54%	100.00%	96.67%	99.06%

FC, functional connectivity; MST, minimum spanning tree; SAD, social anxiety disorder; MCI, mild cognitive impairment; ADHD, attention deficit hyperactivity disorder; MDD, major depressive disorder; AUC, area under receiver operating characteristic (ROC) curve.

**Table 7:** Classification results of different frequencies.

Frequency		Accuracy	Sensitivity	Specificity	AUC
HC	MDD				
0.29	0.21	97.54%	100.00%	96.67%	99.06%
0.14	0.11	85.90%	92.17%	70.00%	87.98%
0.07	0.06	80.29%	91.50%	62.47%	84.49%

HC, healthy control group; MDD, major depressive disorder group; AUC, area under receiver operating characteristic (ROC) curve.

## Supplementary Materials

Supplementary 1 Supplemental Text S1: Image acquisition.

Supplementary 2 Supplemental Text S2: Frequent subgraph mining algorithm.

Supplementary 3 Supplemental Text S3: Kruskal's algorithm.

Supplementary 4 Supplemental Text S4: Weisfeiler-Lehman algorithm.

Supplementary 5 Supplemental Text S5: The methods and results of other contrast networks.

Supplementary 6 Supplemental Table S1: Results of multiple linear regression analysis between network properties and confounding variables.

Supplementary 7 Supplemental Table S2: All regions of interest (abbreviations and full names).

Supplementary 8 Supplemental Table S3: Number of frequent subgraph edges.

Supplementary 9 Supplemental Figure S1: Sliding window.

Supplementary 10 Supplemental Figure S2: Discrimination of different subgraphs.

Supplementary 11 Supplemental Figure S3: Minimum spanning tree functional connectivities and degree of the corresponding node.

Supplementary 12 Supplemental Figure S4: Subgraphs and connected patterns in HC and MDD groups.

Supplementary 13 Supplemental Figure S5: Discriminative brain regions and corresponding degree.
