# Supplementary material for: Machine-Learning Classifier for Patients with Major Depressive Disorder: Multifeature Approach Based on a High-Order Minimum Spanning Tree Functional Brain Network
Source: Comput Math Methods Med. 2017 Dec 14;2017:4820935. doi: 10.1155/2017/4820935 (PMC5745775; doi:10.1155/2017/4820935)
Supplement: Supplementary 2 — Supplemental Text S2: Frequent subgraph mining algorithm. [file 4820935.f2.docx]

**Supplemental Text S2:Kruskal algorithm**

The main idea of the Kruskal algorithm([Osipov et al., 2009](#_ENREF_1)):Given a connected graph G(V,E) with n vertices. We firstly construct a connected graph with n vertices and no edges, namely S=E, then choose a minimum weight edge in E, if the two vertices of the edge in different connected components, add the edge into T, otherwise it will discard this edges. Reselecting a minimum weight of the edge, repeat the above process until all nodes fall on the same connected component. Algorithm 1 gives the pseudo code Kruskal algorithm.

Algorithm 1 Kruskal algorithm

| Input: G(V,E)  Output: minimum spanning tree of G  1 Sort the edge $\{e_{1},e_{2},\ldots,e_{n}\}\in$E in G according to the weight  2 Initialization set S, S =E  3 Select the shortest edge (U, V) from the S  4 for each edge $e_{1}\in$E do  4 if e_i_ =(v_1_,v_2_)is not in the same tree, then  5 Join (v_1_,v_2_) in the spanning tree edge set E'  6 end  7 if all the vertices v_i_ contain in E’  8 Break  9 end  10 end  11 E’ is the minimum spanning tree |
| --- |

Osipov, V., Sanders, P., Singler, J., 2009. The Filter-Kruskal Minimum Spanning Tree Algorithm, The Workshop on Algorithm Engineering & Experiments, pp. 52-61.
