# Supplementary material for: Machine-Learning Classifier for Patients with Major Depressive Disorder: Multifeature Approach Based on a High-Order Minimum Spanning Tree Functional Brain Network
Source: Comput Math Methods Med. 2017 Dec 14;2017:4820935. doi: 10.1155/2017/4820935 (PMC5745775; doi:10.1155/2017/4820935)
Supplement: Supplementary 3 — Supplemental Text S3: Kruskal's algorithm. [file 4820935.f3.docx]

**Supplemental Text S3. The concept of frequent subgraph mining and the technology used by gSpan**

Before giving the details of gSpan, there are some preliminaries used to derive the gSpan algorithm ([Yan and Han, 2002](#_ENREF_1)) for frequent subgraph mining.

Definition 1 (Labeled Undirected Graph)

Let *G = (V, E, L, l)* be a labeled undirected graph, where *V* is a set of nodes and $E\subseteq V\times V$ *·V* is a set of edges. *e = {u, v}* indicates an edge between the nodes *u* and *v*. *L* is a set of labels, and *l* is a mapping function that assigns labels to vertices in *V* and edges in *E*.

Definition 2 (Subgraph)

For two labeled undirected graphs, $G_{S}=(V_{S},E_{S},L_{S},l_{S})$ and *G = (V, E, L, l)*, $G_{S}$ is a subgraph of *G* if $V_{S}\subseteq V$,$E_{S}\subseteq E$,$L_{S}\subseteq L$,$l_{s}\subseteq l$.

Definition 3 (Graph Isomorphism)

A graph $G_{1}=(V_{1},E_{1},L_{V1},L_{E1},l_{1})$ is isomorphic to another graph $G_{2}=(V_{2},E_{2},L_{V2},L_{E2},l_{2})$, if and only a bijection *f :*$V_{1}\to V_{2}$ exists such that

*(i）*$\forall u\in V_{1},l_{1}\left( u \right)=l_{2}(f(u))$*,*

*(ii)*$\forall(u,v)\in E_{1}\Longleftrightarrow(f\left( u \right),f(v))\in E_{2}$*,*

*(iii)*$\forall\left( u,v \right)\in E_{1},l_{1}\left( u,v \right)=l_{2}(f\left( u \right),f(v))$*,*

The bijection *f* is an isomorphism between *G1* and *G2*.

Definition 4 (Frequent Subgraph)

Given a set of graphs, G and a support parameter s, a subgraph $g_{s}$is a frequent subgraph if and only $g_{s}$ exists in at least $S\cdot|G|$ of the input graph set.

Definition 5(Intersect-graph)

Given two graphs$G_{1}=(V_{1},E_{1})$and$G_{2}=(V_{2},E_{2})$, the intersect-graph$G^{'}=(V^{'},E^{'})$ is defined as $E^{'}=E_{1}\cap E_{2}$

**DFS lexicographic order.**

This section describes how the gSpan algorithm maps each graph into a unique minimum depth-first search DFS code. We introduce several techniques used by gSpan to mine frequent subgraphs, including depth-first search code, depth first search dictionary, and minimum depth-first search.

In graph *G*, The node *v_i_* is discovered before node *v_j_* if i <j. The gSpan algorithm converts the graphs into a tree form using the depth-first search algorithm.For the DFS tree, all the edges in the DFS tree are called forward-edge, and the edges that are not in the DFS tree are called backward edge. A linear order$\prec_{T}$, is built among all the edges in graph *G* by the following rules (assume $e_{1}=\left( i_{1},j_{1} \right),e_{2}=(i_{2},j_{2})$)

(1) if $i_{1}=i_{2}$and$j_{1}<j_{2}$, ${e_{1}\prec}_{T}e_{2}$

(2)if $i_{1}＜i_{2}$ and $j_{1}=j_{2}$ ， ${e_{1}\prec}_{T}e_{2}$;

(3)if ${e_{1}\prec}_{T}e_{2}$ and${e_{2}\prec}_{T}e_{3}$，${e_{1}\prec}_{T}e_{3}$

Then, an edge can be simply represented by a five-tuple $(i,j,l_{i},l_{\left( i,j \right)},l_{j})$, In this

study, $l_{i}$and$l_{j}$ are the labels of *V_i_* and *V_j_*, respectively, and $l_{\left( i,j \right)}$ is the label of the

edge $（V_{i}{,V}_{j}）$. The edge (0,1) in Fig 1.b can be expressed as (0,1, X, b, Y). The table (b)(c)(d) column in Fig 1. gives the depth-first Search Coding.


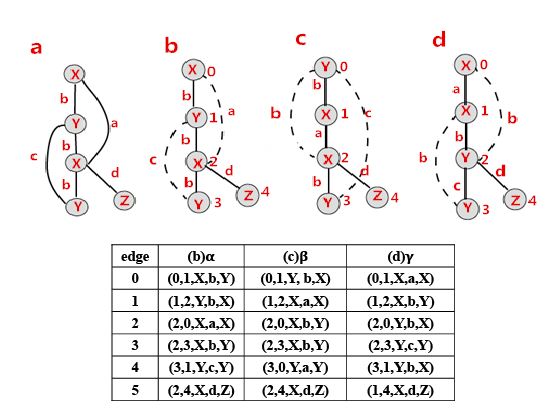


**Fig.1.** Depth-first search tree and its corresponding depth-first search code

Definition 7 (depth-first search [DFS] code)

Given a DFS tree *T* for a graph *G*, an edge sequence $（e_{i}）$can be constructed based

on *T*, such that$e_{i}\prec Te_{i+1}$, where $i=0,\ldots,\left| E \right|-1$,$（e_{i}）$ is called a DFS code,

denotedas code (G,T).

Definition 8: (depth-first search dictionary order)

Given two depth-first search sequences,$a=(a_{0},a_{1},\ldots,a_{m})$and$\beta=(\beta_{0},\beta_{1},\ldots,\beta_{m})$

If the following condition is satisfied,then;$\alpha\leq\beta$:

(i)$\exists t,0\leq t\leq\min\left( m,n \right),a_{k}=\beta_{k} for k<t,a_{t}\prec_{e}\beta_{t}$

(ii)$a_{k}=\beta_{k} for 0\leq k\leq m,n\geq m$

In graph *G*, the minimum one of all the DFS lexicographic order is called Minimum DFS Code of G. Fig. 2 shows a DFS code tree, where all the minimum DFS codes of frequent subgraphs can be discovered through DFS of the code tree. It is noteworthy that the red nodes contain the same subgraph with different DFS codes, but $g'$ is not the minimum DFS code, so the whole branch of $g'$can be pruned because it will not contain any minimum DFS code


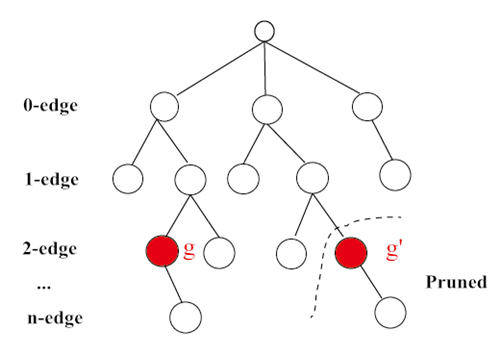


**Fig.2.** DFS code tree.

The idea of the gSpan algorithm ([Yan and Han, 2002](#_ENREF_1))is first to construct a lexicographic order in the diagram, matching each graph to a unique minimal DFS code as a typical label. Then, based on the dictionary order gSpan, the depth-first search strategy is used to mine the frequent connected subgraph model. In this study, the hierarchical search space of frequent subgraphs is called the DFS code tree, and each node in the tree represents a DFS code. *K + 1* layer subgraphs are generated by adding a frequent edge to the K-level subgraph. Finally, all subgraphs that are not the smallest DFS series will be subtracted from the generation of redundant candidate subsets. Algorithm 1 gives the pseudo-code of the gSpan algorithm.

**Algorithm 1**.frequent subgraph mining algorithm

| Input: G  Output: frequent subgraph S  1 Sort the labels in G according to the frequency;  2 Remove infrequent vertices and edges;  3 Relabel the remaining vertices and edges;  4 S^1^← all frequent 1-edge graphs in G;  5 Sort S^1^ in DFS lexicographic order;  6 S ← S^1^;  7 for each edge e∈ S^1^ do  8 Initialize s with e, set s.G by graphs which contain e;  9 if s ≠min(s) then  10 return;  11 end  12 S ←S U s;  13 Enumerate s in each graph in G and count its children;  14 for each c, c is s’child do  15 if support(c)≥ minSup then  16 s←c;  17 end  18 Go to step 9;  19 G←G -e;  20 if \|G\| ≤ minSup then  21 Break;  22 end  23 end  24 end |
| --- |

Yan, X., Han, J., 2002. gSpan: Graph-Based Substructure Pattern Mining, IEEE International Conference on Data Mining, p. 721.
