# Supplementary material for: Machine-Learning Classifier for Patients with Major Depressive Disorder: Multifeature Approach Based on a High-Order Minimum Spanning Tree Functional Brain Network
Source: Comput Math Methods Med. 2017 Dec 14;2017:4820935. doi: 10.1155/2017/4820935 (PMC5745775; doi:10.1155/2017/4820935)
Supplement: Supplementary 4 — Supplemental Text S4: Weisfeiler-Lehman algorithm. [file 4820935.f4.docx]

**Supplemental Text S4. Weisfeiler-Lehman algorithm**

Given two graphs *G* and *H*, let $\sum_{0}$be the original set of node labels of *G* and *H*, and $\sum_{i}$ be the set of letters that occur as node labels at least once in G or H at the end of
the *i*-th iteration of the Weisfeiler-Lehman algorithm. Assume that all $\sum_{i}=\{\sigma_{i1},\sigma_{i2},\ldots,\sigma_{i|\sum_{i}|}\}$ are pairwise disjointed. The Weisfeiler-Lehman subtree kernel ([Shervashidze et al., 2011](#_ENREF_1)) with h iterations of *G* and *H* is defined as follows:

$$k^{h}\left( G,H \right)=<\emptyset^{h}\left( G \right),\emptyset^{h}(H)>$$

$$\emptyset^{h}\left( G \right)=(C_{0}\left( G,\sigma_{01} \right),\ldots,C_{0}\left( G,\sigma_{0\left| \sum_{0} \right|} \right),\ldots,C_{h}\left( G,\sigma_{h1} \right),\ldots,C_{h}(G,\sigma_{h|\sum_{h}|})$$

$$\emptyset^{h}\left( H \right)=(C_{0}\left( H,\sigma_{01} \right),\ldots,C_{0}\left( H,\sigma_{0\left| \sum_{0} \right|} \right),\ldots,C_{h}\left( H,\sigma_{h1} \right),\ldots,C_{h}(H,\sigma_{h|\sum_{h}|})$$

Where $C_{i}\left( G,\sigma_{ij} \right)$and $C_{i}\left( H,\sigma_{ij} \right)$ respectively denote the number of occurrences of the node label $\sigma_{ij}$ after the iterations of *G* and *H*.


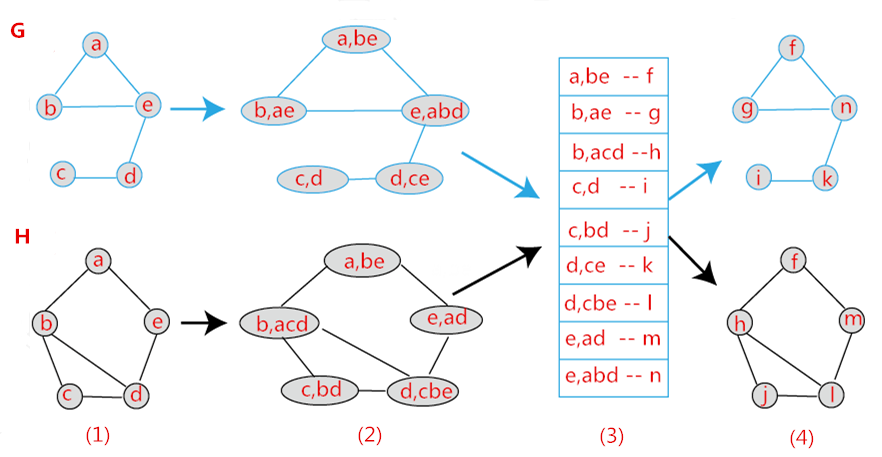


**Fig.1**. WL subtree kernel construction process

**Fig.1.** Illustration of the construction process of the WL subtree kernel. For given two networks *G* and *H*, (1) the initial labeled networks, (2) augmented labels, (3) label compression, (4) relabeled networks. If the iteration time is set as 1, the label set is L={a,b,c,d,e,f,g,h,i,j,k,l,m,n}$\phi\left( G \right)=\left\{ 1,1,1,1,1,1,1,0,1,0,1,0,0,1 \right\},\phi\left( H \right)=\{1,1,1,1,1,1,0,1,0,1,0,1,1,0\}$

So, the $k\left( G,H \right)=<\phi\left( G \right),\phi\left( H \right)>=6$

The main idea of Weisfeiler-Lehman subgraph isomorphism test : if those two graphs are unlabeled (i.e., vertices of the graph have not been assigned labels), first label each vertex with the number of edges that are connected to that vertex. Then, at each iteration step, the label of each vertex is updated based on its previous label and the labels of its neighbors. That is, compress the sorted set of updated node labels of each vertex into a new and shorter label. This process iterates until the node label sets are identical, or the number of iteration reaches its predefined maximum value.The algorithm 1 is the pseudo code of the Weisfeiler-Lehman test of graph isomorphism of the iteration is 1-dim.

**Algorithm 1**: One iteration of the 1-dim. Weisfeiler-Lehman test of graph isomorphism.

| 1 Step 1:Multiset-label determination  2 For i=0,set $M_{i}\left( v \right)=l_{0}\left( v \right)=l(v)$  3 For i>0,Assign a Multiset-label $M_{i}(v)$ to each node v in G and G’ which consists of the  Multiset $\{l_{i-1}\left( u \right)\vert u\in N\left( v \right)\}$  4 Step 2:Sorting each multiset  5 Sort elements in $M_{i}\left( v \right)$ in ascending order and concatenate them into a string $s_{i}\left( v \right)$  6 Add $l_{i-1}\left( v \right)$ as a prefix to $s_{i}\left( v \right)$ and call the resulting string $s_{i}\left( v \right)$  7 Step 3:Label compression  8 Sort all of the strings $s_{i}\left( v \right)$ for all v from G and G’ in ascending order  9 Use a function $f:\Sigma^{*}=\Sigma$ Map each string $s_{i}\left( v \right)$ to a new compressed label.  10 Step 4:Relabeling  11 Set $l_{i}\left( v \right):=f(s_{i}(v))$ for all nodes in G and G’ |
| --- |

Shervashidze, N., Schweitzer, P., Van Leeuwen, E.J., Mehlhorn, K., Borgwardt, K.M., 2011. Weisfeiler-Lehman Graph Kernels. Journal of Machine Learning Research 12, 2539-2561.
