# Supplementary material for: Machine-Learning Classifier for Patients with Major Depressive Disorder: Multifeature Approach Based on a High-Order Minimum Spanning Tree Functional Brain Network
Source: Comput Math Methods Med. 2017 Dec 14;2017:4820935. doi: 10.1155/2017/4820935 (PMC5745775; doi:10.1155/2017/4820935)
Supplement: Supplementary 5 — Supplemental Text S5: The methods and results of other contrast networks. [file 4820935.f5.docx]

**Supplemental Text S5. The methods and results of other contrast networks**

**Pearson correlation network**

*Node definition:* In the current study, a prior atlas of automated anatomical labeling was used to define the nodes. The brain was divided into 90 regions (45 for each hemisphere), with each region representing a node in the network. The mean time series for all voxels in each region was calculated as part of the time series of the corresponding node.

*Edge definition:* We used Pearson correlation coefficient to calculate the correlation coefficient of average time series between any two nodes. The mathematical definitions follows below:

$r=\frac{\sum_{i=1}^{n} (X_{i}-\overline{X})(Y_{i}-\overline{Y})}{\sqrt{\sum_{i=1}^{n} (X_{i}-\overline{X})^{2}}\sqrt{\sum_{i=1}^{n} (Y_{i}-\overline{Y})^{2}}}$ (1)

X_i_ and Y_i_ respectively represent time series of voxel i and j. We then generated a N × N time series correlation matrix. Here, N is the number of node in the given parcellation.

*Threshold selection criterions:* In the current study, sparsity, S, was used to set the threshold, which was defined as the ratio of the number of real existing edges divided by the number of maximum possible edges in the network. The selected threshold ensured that all networks from different groups had the same number of nodes and edges, which are the two golden rules for comparing complex networks [[1](#_ENREF_1)]. Previous studies have reported that resting state functional brain networks exhibit the typical features of small-worldness in both MDD patients and healthy controls (for review, see [[2](#_ENREF_2)]). To determine a data-specific small-world regime, each network was thresholded over a wide sparsity space with the following rules:

a) the average degree of all nodes is greater than 2×Lg (N), N = 90. N is the number of nodes in the network.

b) the small-worldness scalar σ> 1.1.

*Selected features:* Unless otherwise mentioned, all formulas introduced below are based on *G = (N,K)*, a network *G* with *N* nodes and *K* edges. Three nodal measures were examined in the current study: degree (*k_i_*), betweenness centrality (*b_i_*) and nodal efficiency (*e_i_*).

Degree*.* Formally, the degree of node *i* is defined as:

$k_{i}=\sum_{j\in G} a_{ij}$ (2)

where *a_ij_* is the element (*i,j*) in the network. Degree is a simple measurement of connectivity of a node with the rest of nodes in a network.

Nodal efficiency. The nodal efficiency of node *i* is computed as [[3](#_ENREF_3)]:

$e_{i}=\frac{1}{N-1}\sum_{j\neq i\in G} d_{ij}$ (3)

where *d_ij_* is the shortest path length between node *i* and node *j* in *G*. The shortest path length is the minimum number of edges for the network among all possible paths from one node to another in *G*. Nodal efficiency measures the ability of information propagation between a given node *i* with the rest of nodes in a network.

Betweenness Centrality. The betweenness centrality of node is measured as [[4](#_ENREF_4)]:

$b_{i}=\sum_{m\neq i\neq n\in G} \frac{\sigma_{mn}(i)}{\sigma_{mn}}$ (4)

where *σ_mn_* is the total number of shortest paths (paths with the shortest path length) from node *m* to node *n*, and *σ_mn_(i)* is the number of shortest paths from node *m* to node *n* that pass through the node *i*. Betweenness centrality of a node captures the influence of the node over information flow between all the other nodes in the network.

*Abnormal Brain Regions:*

Table 1. Abnormal Brain Regions detected by Pearson correlation network

| Brain Regions | P-Value | | |
| --- | --- | --- | --- |
|  | Degree | Betweenness Centrality | Nodal Efficiency |
| MDD<Control | | | |
| Left fusiform gyrus | **0.016** | 0.151 | **0.047** |
| Left inferior frontal gyrus, orbital part | **0.017** | 0.179 | **0.034** |
| Right cuneus | **0.025** | **0.001** | 0.093 |
| Right superior frontal gyrus, medial | **0.035** | 0.495 | 0.151 |
| Right middle frontal gyrus, orbital part | **0.049** | 0.557 | **0.025** |
| Left inferior frontal gyrus, opercular part | **0.050** | 0.295 | 0.141 |
| Left calcarine fissure and surrounding cortex | 0.058 | 0.547 | **0.046** |
| Right inferior frontal gyrus, opercular part | 0.063 | **0.048** | 0.178 |
| Right superior frontal gyrus, medial orbital | 0.082 | 0.848 | **0.044** |
| MDD>Control | | | |
| Right hippocampus | **0.001** | **0.005** | **0.003** |
| Left angular gyrus | **0.008** | 0.284 | **0.011** |
| Right posterior cingulate gyrus | **0.008** | 0.167 | 0.007 |
| Right thalamus | **0.008** | **0.004** | **0.008** |
| Right lenticular nucleus, putamen | **0.014** | 0.213 | **0.023** |
| Right middle occipital gyrus | **0.021** | 0.443 | **0.027** |
| Right median cingulate and paracingulate gyri | 0.070 | 0.482 | **0.014** |
| Left median cingulate and paracingulate gyri | 0.073 | 0.632 | **0.048** |

Regions were considered abnormal in MDD patients if they exhibited significant between-group differences (p < 0.05, uncorrected) in at least one of the three nodal metrics (bold font).

**Partial correlation method**

*Node definition:* The same way with Pearson method.

*Edge definition:* We used Partial correlation coefficient to calculate the correlation coefficient of average time series between any two nodes. The mathematical definitions follows below:

$\hat{\rho}_{XY\cdot Z}=\frac{N\sum_{i=1}^{N} e_{X,i}e_{Y,i}-\sum_{i=1}^{N} e_{X,i}\sum_{i=1}^{N} e_{Y,i}}{\sqrt{N\sum_{i=1}^{N} e_{X,i}^{2}-\left( \sum_{i=1}^{N} e_{X,i} \right)^{2}}\sqrt{N\sum_{i=1}^{N} e_{Y,i}^{2}-\left( \sum_{i=1}^{N} e_{Y,i} \right)^{2}}}$ (5)

*Threshold selection criterions:* The same way with Pearson method.

*Selected features:* The same way with Pearson method.

*Abnormal Brain Regions:*

Table 2. Abnormal Brain Regions detected by Partial correlation network

| Brain Regions | P-Value | | |
| --- | --- | --- | --- |
|  | Degree | Betweenness Centrality | Nodal Efficiency |
| MDD<Control | | | |
| Left thalamus | **0.001** | **0.009** | **0.008** |
| Left inferior frontal gyrus, orbital part | **0.007** | **0.005** | 0.192 |
| Right inferior frontal gyrus, triangular part | **0.035** | 0.146 | **0.005** |
| Left median cingulate and paracingulate gyri | **0.009** | 0.103 | 0.343 |
| Right inferior occipital gyrus | **0.011** | 0.122 | 0.630 |
| Right amygdala | **0.014** | 0.112 | 0.162 |
| Left superior occipital gyrus | **0.015** | **0.049** | 0.232 |
| Right olfactory cortex | 0.436 | 0.298 | **0.019** |
| Left temporal pole: middle temporal gyrus | **0.022** | 0.086 | 0.191 |
| Left inferior frontal gyrus, opercular part | **0.045** | 0.093 | 0.025 |
| Left superior frontal gyrus, orbital part | **0.046** | 0.069 | 0.672 |
| MDD>Control | | | |
| Right inferior frontal gyrus, orbital part | 0.197 | 0.026 | **0.009** |
| Right parahippocampal gyrus | **0.015** | 0.232 | **0.046** |
| Left superior frontal gyrus, dorsolateral | **0.020** | 0.179 | 0.055 |
| Right precentral gyrus | **0.025** | 0.390 | 0.097 |
| Right lenticular nucleus, putamen | 0.098 | **0.043** | **0.025** |
| Left superior parietal gyrus | **0.028** | 0.225 | 0.214 |
| Right gyrus rectus/straight gyrus | **0.037** | 0.653 | 0.050 |
| Left amygdale | 0.149 | 0.768 | **0.037** |
| Right temporal pole: superior temporal gyrus | **0.448** | **0.035** | 0.874 |
| Left superior frontal gyrus, orbital part | **0.045** | 0.147 | 0.404 |
| Right lenticular nucleus, pallidum | **0.045** | 0.172 | 0.098 |

**High-order** **functional connectivity network without MST analysis**

The process framework is consistent with the proposed method except that the MST analysis is not performed.

.*Abnormal functional connectivity:*

Table 3. Abnormal connectivity detected by high-order network

| Functional Connectivity | | P-Value | |  |
| --- | --- | --- | --- | --- |
| ROI A | ROI B | Degree | Betweenness Centrality | Nodal Efficiency |
| IOG.L | ANG.L | **0.004** | **0.009** | **0.007** |
| SMA.L | STG.R | **0.004** | **0.010** | **0.043** |
| SFGmed.L | TPOmid.L | **0.009** | **0.032** | **0.003** |
| PreCG.L | ROL.L | **0.014** | **0.033** | 0.220 |
| AMYG.L | CAU.L | **0.014** | 0.091 | 0.140 |
| IPL.R | PreCG.R | **0.022** | **0.022** | 0.243 |
| PUT.R | HES.R | **0.023** | **0.049** | 0.800 |
| INS.R | CUN.R | 0.140 | **0.031** | **0.029** |
| CUN.L | PreCG.R | **0.029** | 0.102 | 0.078 |
| INS.R | DCG.R | 0.208 | **0.032** | 0.094 |
| PreCG.R | IFGtriang.L | **0.033** | 0.089 | 0.278 |
| IOG.L | REC.R | **0.038** | 0.095 | 0.562 |
| SPG.L | ITG.R | **0.040** | 0.123 | 0.058 |
| ORBmid.R | MTG.R | **0.047** | 0.168 | 0.464 |
| PUT.R | HES.R | 0.051 | **0.006** | 0.058 |
| TPOsup.L | PAL.R | 0.066 | **0.013** | 0.066 |
| SMA.R | IPL.R | 0.104 | 0.104 | **0.026** |
| DCG.R | TPOmid.R | 0.491 | 0.116 | **0.035** |
| LING.L | IFGoperc.R | 0.231 | **0.035** | 0.175 |
| SMA.L | PreCG.R | 0.231 | **0.039** | 0.521 |
| SOG.L | PHG.R | **0.045** | 0.083 | 0.584 |
| CAU.L | SPG.R | 0.093 | **0.006** | 0.348 |
| HES.L | OLF.R | **0.017** | 0.158 | 0.211 |
| SMA.L | IFGtriang.R | 0.074 | 0.322 | **0.019** |
| SMA.L | IPL.L | **0.045** | **0.023** | 0.058 |
| INS.R | DCG.R | **0.035** | 0.129 | 0.175 |
| PreCG.R | IFGtriang.L | **0.004** | **0.015** | **0.027** |
| IOG.L | REC.R | **0.006** | **0.014** | 0.056 |
| SPG.L | ITG.R | **0.014** | **0.030** | **0.008** |
| ORBmid.R | MTG.R | **0.036** | 0.135 | **0.008** |
| INS.R | SPG.R | **0.017** | **0.025** | 0.053 |
| STG.L | SPG.R | **0.030** | 0.067 | **0.025** |
| STG.L | IPL.R | **0.033** | 0.552 | **0.039** |
| STG.L | FFG.R | 0.193 | 0.207 | **0.004** |
| ORBmid.L | IPL.L | **0.039** | **0.034** | **0.044** |

**References:**

1. Bollobás, B., *Random graphs*. Vol. 73. 2001: Cambridge university press.

2. Bullmore, E.T. and D.S. Bassett, *Brain graphs: graphical models of the human brain connectome.* Annual review of clinical psychology, 2011. **7**: p. 113-140.

3. Achard, S. and E. Bullmore, *Efficiency and cost of economical brain functional networks.* PLoS Computational Biology, 2007. **3**(2): p. e17.

4. Freeman, L.C., *A set of measures of centrality based on betweenness.* Sociometry, 1977: p. 35-41.
