# Supplementary material for: Machine-Learning Classifier for Patients with Major Depressive Disorder: Multifeature Approach Based on a High-Order Minimum Spanning Tree Functional Brain Network
Source: Comput Math Methods Med. 2017 Dec 14;2017:4820935. doi: 10.1155/2017/4820935 (PMC5745775; doi:10.1155/2017/4820935)
Supplement: Supplementary 6 — Supplemental Table S1: Results of multiple linear regression analysis between network properties and confounding variables. [file 4820935.f6.docx]

**Supplemental Table 1:Results of multiple** **linear regression analysis between network properties and confounding variables**

| Confounding Variables | Coefficients | Std. Error | T Stat. | | P | Lower 95% | Upper 95% |
| --- | --- | --- | --- | --- | --- | --- | --- |
| Betweenness (Adj. R_sqr_ = -0.013_,_ P = 0.421) | | | | | | | |
| Intercept | 151962.487 | 17376.918 | 8.745 | 2.323 | | 117215.191 | 186709.783 |
| Gender | 11342.125 | 6544.755 | 1.733 | 0.088 | | -1744.922 | 24429.173 |
| Age | 62.277 | 350.968 | 0.177 | 0.859 | | -639.527 | 764.082 |
| Educational Attainments | 1342.041 | 2456.227 | 0.546 | 0.586 | | -3569.490 | 6253.571 |
| Eccentricity(Adj. R_sqr_ = -0.007_,_ P = -0.243) | | | | | | | |
| Intercept | 78.928 | 8.729 | 9.042 | <0.001 | | 61.473 | 96.383 |
| Gender | 3.727 | 3.288 | 1.134 | 0.261 | | -2.846 | 10.301 |
| Age | 0.055 | 0.176 | 0.313 | 0.755 | | -0.297 | 0.408 |
| Educational Attainments | 1.034 | 1.234 | 0.838 | 0.405 | | -1.432 | 3.501 |

The range of age is 17–51 years. Optional values of gender are male and female. Optional values of educational attainments are illiteracy, primary school, junior high school, senior high school, junior college, college, graduate degree and above. Adj. R_sqr_, adjusted R square. Coefficients, regression coefficient. Std. Error, standard error. T stat., T statistic. Lower 95%, low bound of 95% confidence limits. Upper 95%, upper bound of 95% confidence limits
