# Supplementary material for: Machine-Learning Classifier for Patients with Major Depressive Disorder: Multifeature Approach Based on a High-Order Minimum Spanning Tree Functional Brain Network
Source: Comput Math Methods Med. 2017 Dec 14;2017:4820935. doi: 10.1155/2017/4820935 (PMC5745775; doi:10.1155/2017/4820935)
Supplement: Supplementary 7 — Supplemental Table S2: All regions of interest (abbreviations and full names). [file 4820935.f7.docx]

**Supplemental Table T2. The abbreviation and full name of the regions of interest**

| Abbreviation | Full Name |
| --- | --- |
| ACG.L | Left Anterior cingulate and paracingulate gyri |
| ACG.R | Right Anterior cingulate and paracingulate gyri |
| AMYG.L | Left Amygdala |
| AMYG.R | Right Amygdala |
| CAL.L | Left Calcarine ﬁssure and surrounding cortex |
| CAU.L | Left Caudate nucleus |
| CAU.R | Right Caudate nucleus |
| CUN.L | Left Cuneus |
| CUN.R | RightCuneus |
| DCG.L | Left Median cingulate and paracingulate gyri |
| DCG.R | Right Median cingulate and paracingulate gyri |
| HES.L | Left Heschl gyrus |
| HIP.L | Left Hippocampus |
| HIP.R | Right Hippocampus |
| IFGtriang.L | Left Inferior frontal gyrus, triangular part |
| IFGtriang.R | Right Inferior frontal gyrus, triangular part |
| IFGoperc.L | Inferior frontal gyrus, opercular part |
| IFGoperc.R | Inferior frontal gyrus, opercular part |
| ITG.R | Right Inferior temporal gyrus |
| LING.L | Left Lingual gyrus |
| LING.R | Right Lingual gyrus |
| MFG.L | Left Middle frontal gyrus |
| MTG.R | Right Lingual gyrus |
| ORBinf.L | Left Inferior frontal gyrus, orbital part |
| ORBinf.R | Right Inferior frontal gyrus, orbital part |
| ORBmid.L | Left Middle frontal gyrus, orbital part |
| ORBmid.R | Right Middle frontal gyrus, orbital part |
| ORBsup.L | Left Superior frontal gyrus, orbital part |
| ORBsup.R | Right Superior frontal gyrus, orbital part |
| PAL.L | Left Lenticular nucleus, pallidum |
| PAL.R | Right Lenticular nucleus, pallidum |
| PCG.L | Left Posterior cingulate gyrus |
| PCG.R | Right Posterior cingulate gyrus |
| PCL.R | Right Paracentral lobule |
| PCUN.L | Left Precuneus |
| PCUN.R | Right Precuneus |
| PHG.L | Left Parahippocampal gyrus |
| PHG.R | Right Parahippocampal gyrus |
| PoCG.R | Right Postcentral gyrus |
| PreCG.L | Left Precentral gyrus |
| PreCG.R | Right Precentral gyrus; |
| PUT.L | Left Lenticular nucleus, putamen |
| PUT.R | Right Lenticular nucleus, putamen |
| ROL.R | Right Rolandic operculum |
| SFGdor.R | Right Superior frontal gyrus, dorsolateral |
| SFGmed.L | Left Superior frontal gyrus, medial |
| STG.L | Left Superior temporal gyrus |
| THA.L | Left Thalamus |
| THA.R | Right Thalamus |
| TPOmid.L | Left Temporal pole: middle temporal gyrus |
| TPOmid.R | Right Temporal pole: middle temporal gyrus |
