# Supplementary material for: Machine-Learning Classifier for Patients with Major Depressive Disorder: Multifeature Approach Based on a High-Order Minimum Spanning Tree Functional Brain Network
Source: Comput Math Methods Med. 2017 Dec 14;2017:4820935. doi: 10.1155/2017/4820935 (PMC5745775; doi:10.1155/2017/4820935)
Supplement: Supplementary 8 — Supplemental Table S3: Number of frequent subgraph edges. [file 4820935.f8.docx]

**Supplemental Table T3 .The number of frequent subgraph edges**

| HC | | MDD | |
| --- | --- | --- | --- |
| edges | **The number of edges** | **edges** | **The number of edges** |
| 1 | 4005 | 1 | 4005 |
| 2 | 33 | 2 | 47 |
| 3 | 14 | 3 | 26 |
| 4 | 5 | 4 | - |

Where HC is Healthy Controls and MDD is Major Depressive Disorder
