# Supplementary material for: Machine-Learning Classifier for Patients with Major Depressive Disorder: Multifeature Approach Based on a High-Order Minimum Spanning Tree Functional Brain Network
Source: Comput Math Methods Med. 2017 Dec 14;2017:4820935. doi: 10.1155/2017/4820935 (PMC5745775; doi:10.1155/2017/4820935)
Supplement: Supplementary 9 — Supplemental Figure S1: Sliding window. [file 4820935.f9.docx]

**Supplemental Figure S1.Sliding time window**


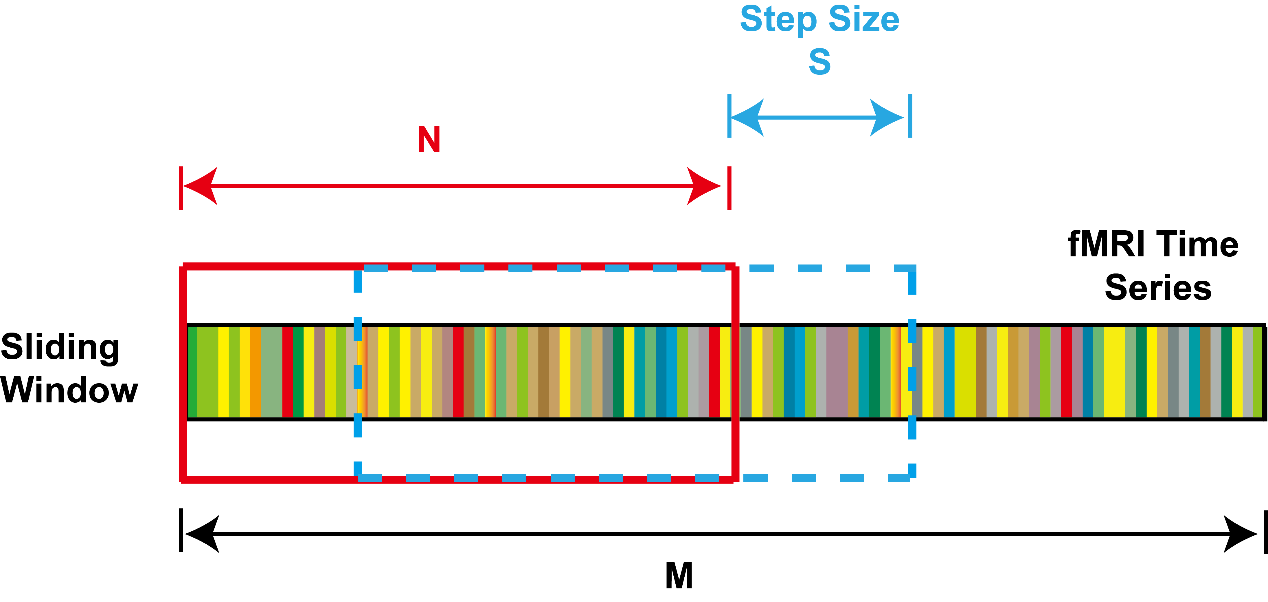


**Figure S1. Illustrates a schematic diagram of a sliding time window,** where the length of the sliding window is *N* and the step size between two successive windows is *S*. *M* is the length of the fMRI time series, and the total number of segments that can be generated by moving the sliding window is $K=\left\lfloor(M-N)/s \right\rfloor+1$.
