# Supplementary material for: Machine-Learning Classifier for Patients with Major Depressive Disorder: Multifeature Approach Based on a High-Order Minimum Spanning Tree Functional Brain Network
Source: Comput Math Methods Med. 2017 Dec 14;2017:4820935. doi: 10.1155/2017/4820935 (PMC5745775; doi:10.1155/2017/4820935)
Supplement: Supplementary 10 — Supplemental Figure S2: Discrimination of different subgraphs. [file 4820935.f10.docx]

**Supplemental Figure S2. The discrimination of different subgraph**

**
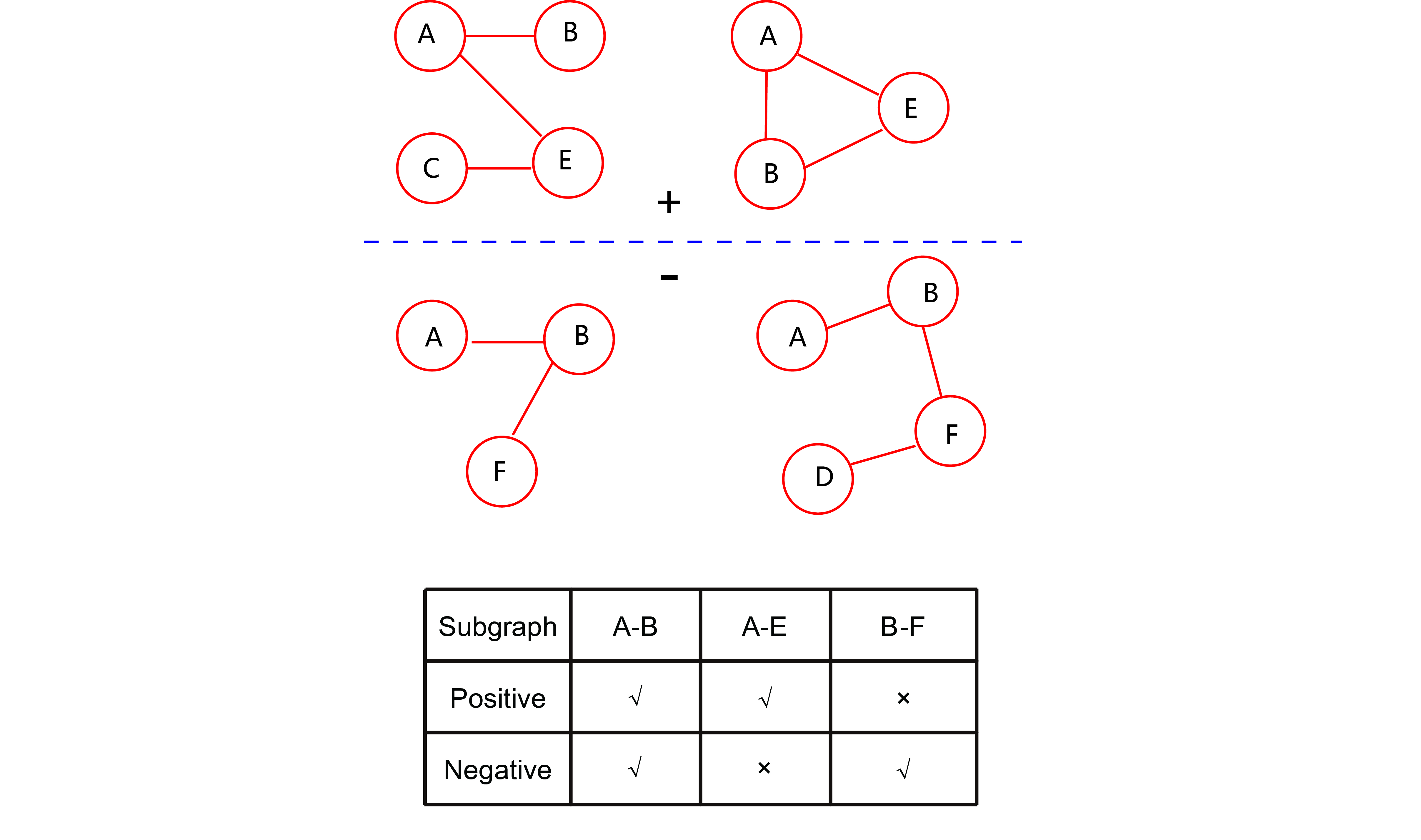
**

**Fig.2. illustrates the discrimination of different subgraph.** The $'A-B'$ both exists in the positive and negative samples, the $'A-E'$ exists only in the positive samples and the negative samples does not exist, and the $'B-F'$ only in the negative samples exists in a positive samples does not exist. Therefore, the subgraph feature $'A-E'$ and$'B-F'$ is more useful than $'A-B'$ and other subgraphs to positive or negative samples.
