# Supplementary material for: Machine-Learning Classifier for Patients with Major Depressive Disorder: Multifeature Approach Based on a High-Order Minimum Spanning Tree Functional Brain Network
Source: Comput Math Methods Med. 2017 Dec 14;2017:4820935. doi: 10.1155/2017/4820935 (PMC5745775; doi:10.1155/2017/4820935)
Supplement: Supplementary 11 — Supplemental Figure S3: Minimum spanning tree functional connectivities and degree of the corresponding node. [file 4820935.f11.docx]

**Supplemental Figure S3. Minimum spanning tree functional connectivities and degree of the corresponding node**


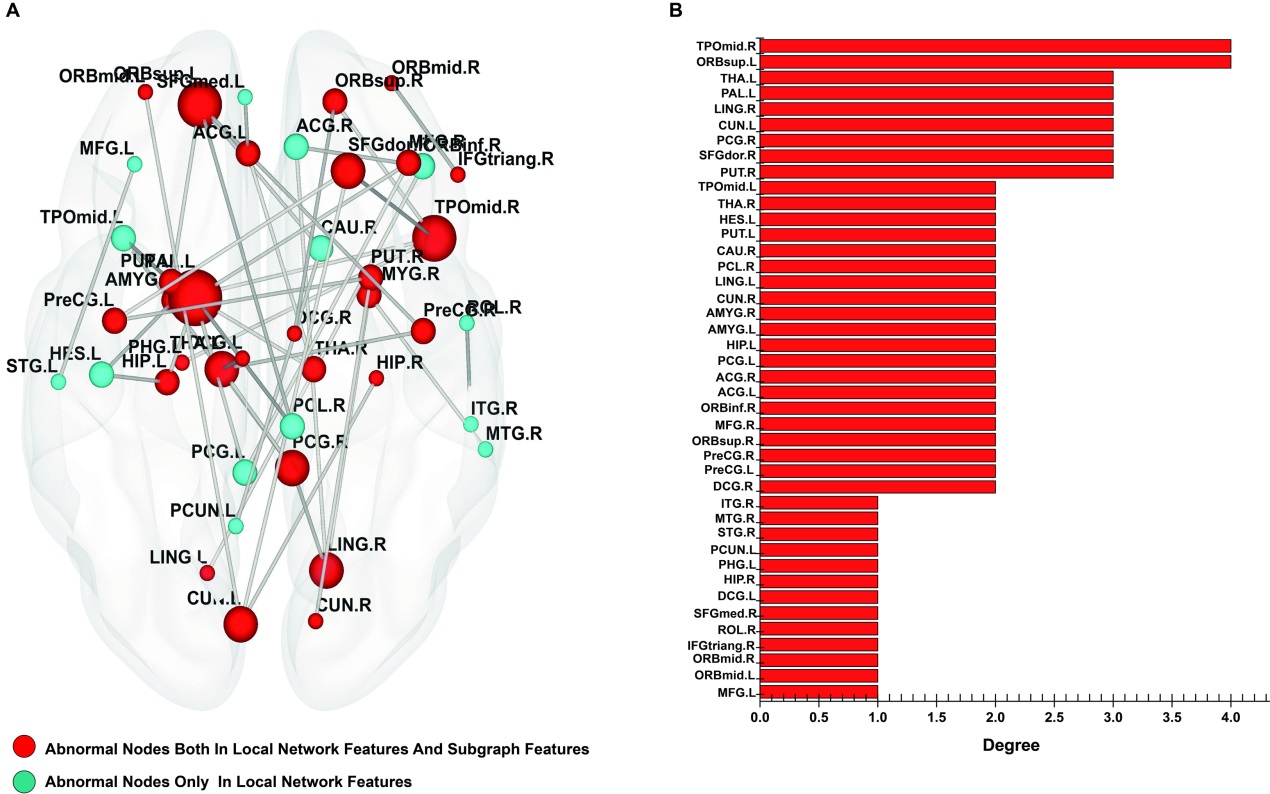


**Figure S3.** **Minimum spanning tree functional connectivities and degree of the corresponding node** (A) Forty functional connectivities of the minimum spanning tree. Red nodes, same nodes as in the minimum spanning tree, subgraphs mined from the HC and MDD groups; blue nodes, those existing only in the minimum spanning tree (not in the subgraphs). Size of nodes represents frequency. (B) Frequency of the nodes involved in the 40 functional connectivities. For all abbreviations for the discriminative brain regions, see Supplemental table S2.
