# Supplementary material for: Machine-Learning Classifier for Patients with Major Depressive Disorder: Multifeature Approach Based on a High-Order Minimum Spanning Tree Functional Brain Network
Source: Comput Math Methods Med. 2017 Dec 14;2017:4820935. doi: 10.1155/2017/4820935 (PMC5745775; doi:10.1155/2017/4820935)
Supplement: Supplementary 12 — Supplemental Figure S4: Subgraphs and connected patterns in HC and MDD groups. [file 4820935.f12.docx]

**Supplemental Figure S4. Subgraphs and connected patterns in HC and MDD groups**


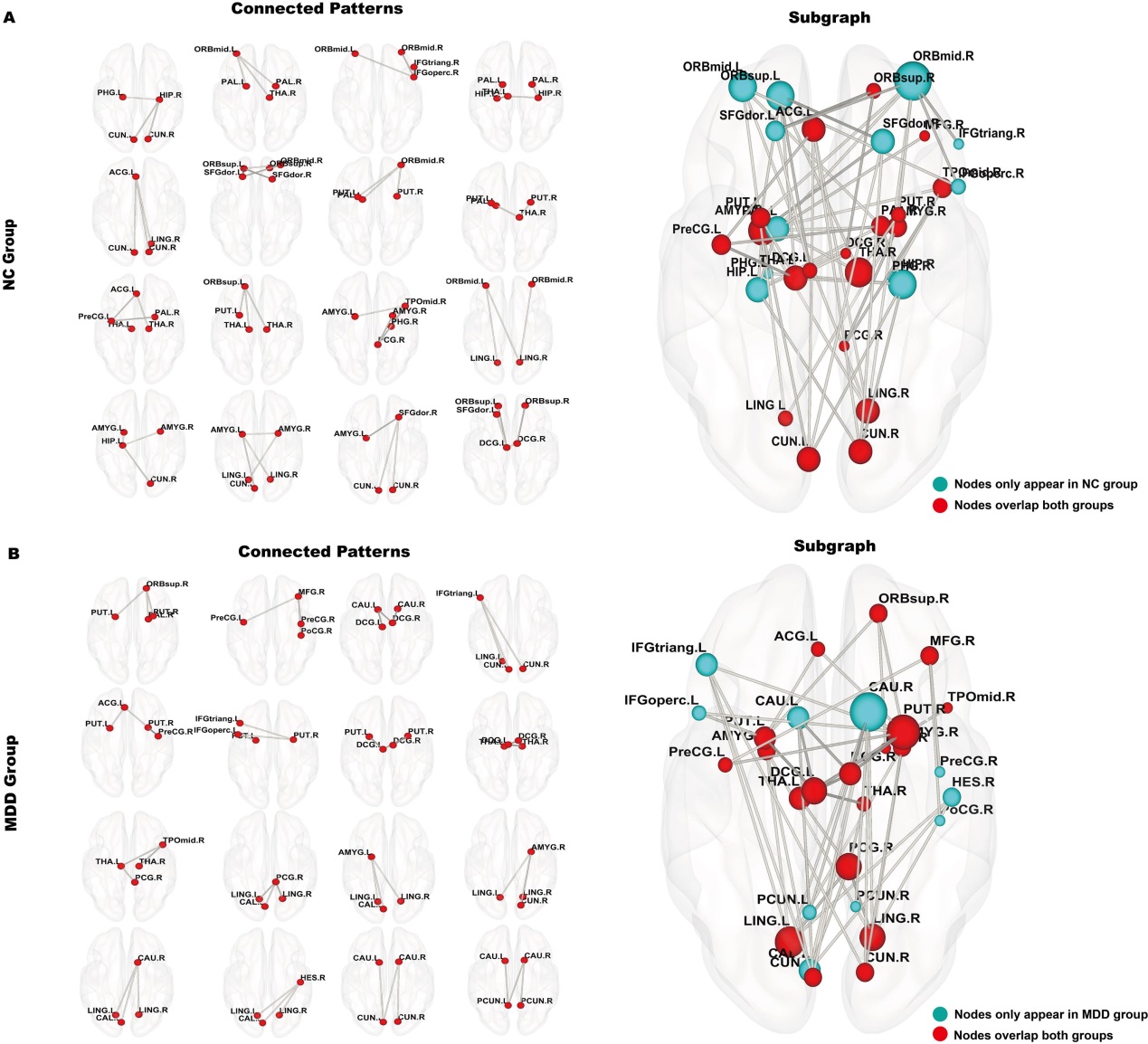


**Figure S4. Subgraphs and connected patterns in HC and MDD groups** ‘Connected patterns’ refers to the 16 discriminative subgraphs of the HC and MDD groups. To analyze these connected patterns, connections in the 16 subgraph connected patterns of the two groups are merged with the ‘Subgraph’ in this figure. Red nodes, those that appear in both subgraphs; blue nodes, those appearing only in one subgraph. Size of each node represents the degree of the node in the subgraphs. For all abbreviations for the discriminative brain regions, see Supplemental table S2.
