# Supplementary material for: Machine-Learning Classifier for Patients with Major Depressive Disorder: Multifeature Approach Based on a High-Order Minimum Spanning Tree Functional Brain Network
Source: Comput Math Methods Med. 2017 Dec 14;2017:4820935. doi: 10.1155/2017/4820935 (PMC5745775; doi:10.1155/2017/4820935)
Supplement: Supplementary 13 — Supplemental Figure S5: Discriminative brain regions and corresponding degree. [file 4820935.f13.docx]

**Supplemental Figure S5. Discriminative brain regions and corresponding degree**


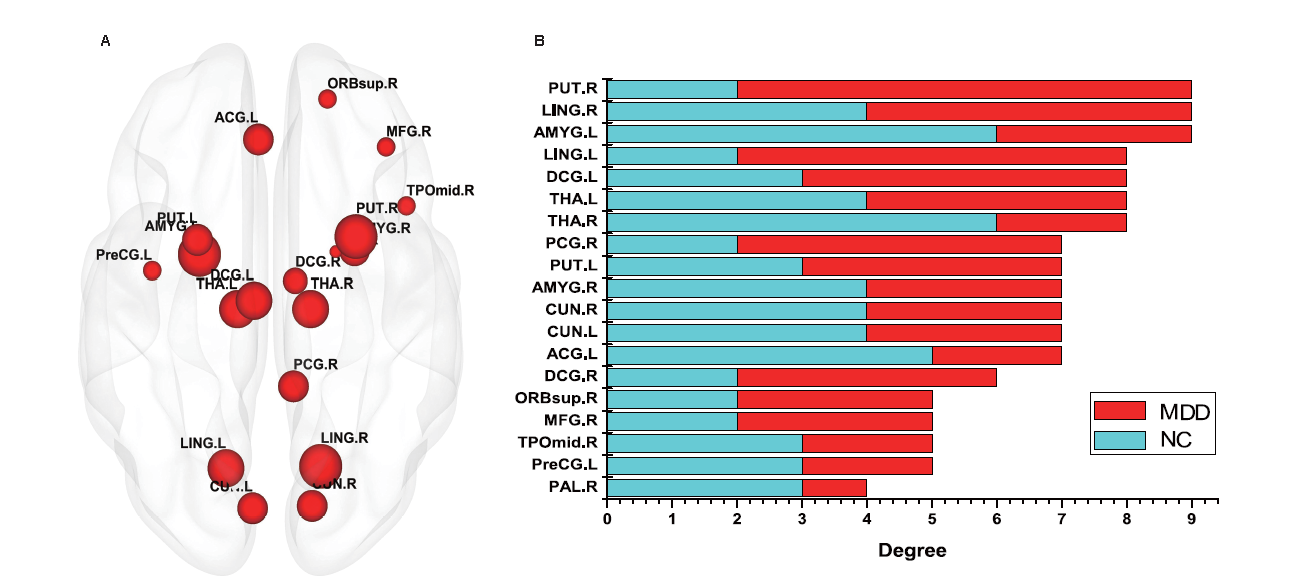


**Figure S5: Discriminative brain regions and corresponding degree** (A) Nodes common to the HC and MDD groups. Size of nodes represents degree. (B) Statistical analysis of the degree of the nodes. For all abbreviations for the discriminative brain regions, see Supplemental table S2.
